# Epigenetics, Resilience, Protective Factors and Factors Promoting Positive Outcomes: A Scoping Review

**DOI:** 10.1002/jdn.70042

**Published:** 2025-10-20

**Authors:** Simone G. Assis, Pedro H. Tavares, Nayara Oliveira, Fernanda Serpeloni, Joviana Q. Avanci

**Affiliations:** ^1^ Department of Studies on Violence and Health Jorge Careli (Claves), National School of Public Health Oswaldo Cruz Foundation Rio de Janeiro Rio de Janeiro Brazil; ^2^ Post Graduate Program in Public Health Oswaldo Cruz Foundation Rio de Janeiro Rio de Janeiro Brazil; ^3^ Genomic Research Institute Central‐West State University (UNICENTRO) Guarapuava Paraná Brazil; ^4^ Department of Studies on Violence and Health Jorge Careli. National School of Public Health Oswaldo Cruz Foundation Rio de Janeiro Rio de Janeiro Brazil

**Keywords:** emotional functioning, epigenetics, resilience, self‐esteem, social support

## Abstract

**Introduction:**

While several studies have examined the relationship between adverse social exposures and epigenetic mechanisms, the association between DNA methylation, resilience, protective factors and factors that promote positive outcomes remains underexplored.

**Goals:**

This study aims to analyse scientific publications on epigenetics, specifically focusing on DNA methylation in relation to resilience and individual/social protective/positive factors.

**Method:**

A scoping review was conducted using the descriptors DNA methylation, resilience, self‐esteem, emotional regulation, social support and social and emotional functioning, covering the years 2008–2019. The databases used included Web of Science, PubMed and Embase. The analysis included 110 articles, reviewed for identification and profile, article focus, objectives, epidemiological and epigenetic methods, protective/positive factors and impacts on physical and mental health.

**Results:**

There has been a significant, gradual increase in publications over the years, particularly regarding epidemiological studies involving human participants. Most studies utilized a candidate gene approach to assess DNA methylation, while broader genome‐wide methylation profiles were less frequently examined. Histone modifications and noncoding RNAs were also discussed, especially in review articles. Resilience was identified as the most studied topic, with analyses focusing on (1) mental disorders, (2) parental mental health, (3) early life stress, (4) biological age and development, (5) clinical and physiological conditions and (6) environmental/socioeconomic factors. A wide variety of genes associating resilience and epigenetics were pointed out, for example, *NR3C1*, *SLC6A4*, *BDNF*, and *FKBP5*. Additionally, individual and social bonds and competencies such as social and emotional functioning, maternal care and social interaction among others were grouped as protective factors or that promote positive outcomes and were linked to genes such as *OXTR*, followed by *FKBP5* and *NR3C1*.

**Discussion:**

The significance of epigenetics in neuroscience and its potential public health applications is still emerging. This field invites reflection on prevention and health promotion strategies and highlights growing evidence that social inequalities, adversities and early‐life experiences may have lasting effects on gene expression throughout the lifespan.

## Graphical abstract

The review shows the connections between DNA methylation, resilience and individual and social protective factors. Resilience emerges as the most extensively studied topic, with a variety of genes identified. Social and emotional functioning, maternal care and social interaction are also investigated, primarily in relation to genes like OXTR.

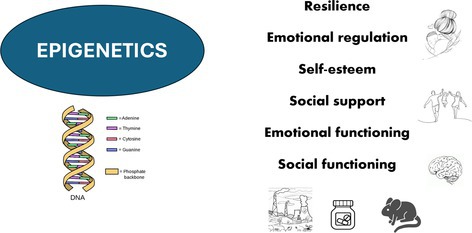

## 1 Introduction

Epigenetics investigates changes in gene expression that do not involve alterations in the DNA sequence. Although these modifications are generally stable over an individual's lifetime, they exhibit remarkable sensitivity to environmental influences, underscoring their dynamic and plastic nature. Increasing interest in health and behavioural sciences through epigenetic mechanisms focuses on how health and disease processes arise from the interaction between genes and the environment, bridging nature and culture. Epigenetic studies enhance our understanding of how environmental factors can ‘enter the skin’ and ultimately influence the functioning of the organism (Maccari et al. 2014; Champagne and Curley 2011).

Epigenetic modifications involve a wide range of phenomena (such as dosage compensation and genomic imprinting) and mechanisms (histone modification, DNA methylation and noncoding RNAs) (Jirtle and Skinner 2007). Among these, DNA methylation is the most extensively investigated and is characterized by a dynamic process that begins with the embryonic development of human beings and continues throughout life. It is the addition of a methyl group to the carbon in position five of cytosine, commonly associated with gene silencing. Changes in DNA methylation may result in total or no change in gene expression or even cause a gradual effect (Burns et al. 2017; Franklin et al. 2012).

Research in social epigenetics has aimed to understand the relationship between adverse social exposures and epigenetic mechanisms, where social inequalities can be expressed at a molecular level (Aristizabal et al. 2020; Burris et al. 2016). In this way, adverse experiences, such as maltreatment, stress in the prenatal period, crime, racism, discrimination and poverty, particularly during the early periods of life, influence DNA methylation patterns (Burns et al. 2017; Franklin et al. 2012; Martin et al. 2022; Cao‐Lei et al. 2014). Biologically, trauma induced by such adversities may produce changes in gene expression through epigenetic modifications such as DNA methylation (Klengel et al. 2014). Epigenome‐wide association studies (*EWAS*) and candidate gene analyses focusing on the impact of adversities in life provide valuable insights into the understanding of the complex pathways involved in different regulatory mechanisms susceptible to stress throughout life. These studies shed light on how environmental risk factors shape gene regulation, potentially leading to phenotypic outcomes (Esposito et al. 2016; Lupien et al. 2009; Teicher et al. 2003; Champagne 2010). Furthermore, they contribute to the understanding of how deleterious social and economic exposures contribute to biomarkers of complex chronic diseases, especially in the context of health inequalities (Vidrascu et al. 2019).

In epidemiology, identifying risk factors for diseases and injuries, especially in psychiatry, has been prioritized (Dudley et al. 2011; Zannas, Provençal, and Binder 2015; Fries et al. 2014; Morgan et al. 2013), and epigenetic changes are increasingly accepted as biomarkers and potential mediators of differential ageing, life expectancy and mental health issues (Horvath and Raj 2018). For example, epigenetic ageing has emerged as a key mechanism linking chronic stress with accelerated ageing and heightened vulnerability to stress‐related disorders (Zannas, Arloth, et al. 2015). However, the specific roles of protective environments and their associated epigenetic mechanisms—those that contribute to the development of resilience and individual and social protective factors—are often overlooked (Smeeth et al. 2021). Numerous studies support the hypothesis that epigenetic differences may distinguish susceptible and resilient individuals (Schiele et al. 2020; Black et al. 2024). Smeeth et al. (2021) highlight that much less is known about how epigenetics is affected by protective factors (e.g., factors that counteract adverse effects) and positive factors (e.g., factors with an independent positive impact) in the environment, and how this contributes to the development of psychological resilience and other individual attributes. Factors that promote positive outcomes involve diverse issues such as well‐being, education and learning, among others. These mechanisms are critical, particularly given the wide variation in individual sensitivity to both adverse and protective/positive experiences (Black et al. 2024).

Resilience has been conceptualized in several ways within literature. Hiebel et al. (2021) identify four waves that explain the development of the concept of resilience over the last 40 years: (1) identifying protective factors that contribute to individual resilience; (2) understanding resilience within developmental and ecological systems; (3) creating interventions and training programs to foster resilience when it is unlikely to occur naturally; and (4) examining multilevel dynamics linking genes, neurobiological development, adaptation, behaviour, context and their specific interactions. Smeeth et al. (2021) note that resilience reflects not simply the absence of risk factors but also the presence of factors that promote positive adaptation.

Despite its widespread use, inconsistencies in the use of the term arise from the nature of potential risk (exposure to stress) and protective/positive processes (individual or environmental level). Although no universal definition exists, Smeeth et al. (2021) synthesized common themes in how researchers use the term resilience in their studies, highlighting notions of positive adaptation despite significant adversity, the ability to rebound from adversity and the presence or absence of good mental or physical health. Exposure to significant adverse events is a central prerequisite for assessing resilience (Hiebel et al. 2021).

Other authors conceptualize resilience as an active and complex construct that involves the interaction between adverse life events and protective/positive factors, both internal and external to the individual, even before birth (Assis et al. 2006). Resilience is also described as a measure of adaptability of a person when faced with adversity (Morgan et al. 2013) or as the absence (or lower index) of psychiatric symptoms compared with individuals exposed to the same events (Jakob et al. 2014). Additionally, resilience is shaped by neurobiological profiles, developmental experiences, cultural and temporal contexts, social interventions and practical training (Kaye‐Kauderer et al. 2021). Protective factors traditionally studied with resilience include emotional regulation (Coutinho et al. 2010), self‐esteem (Coopersmith 1967) and social support (Sherbourne and Stewart 1991).

Resilience and protective/positive factors can be affected throughout the lifespan in at least three ways. First, an initial epigenetic signature may be present from conception or even preconception, determined by genetic variation or inherited epigenetic marks. Second, the epigenome exhibits plasticity, particularly during prenatal development and childhood, making it highly responsive to environmental influences. Third, protective/positive factors present during exposure to adversity can moderate the epigenetic response to stress. Genetic factors also have an important role, directly affecting and moderating environmental impacts on the epigenome. Understanding these mechanisms is essential to uncovering the potential ways in which epigenetic factors contribute to resilience (Smeeth et al. 2021).

Research on epigenetics, resilience and protective/positive factors is highly relevant to public health (Pluess 2017). It emphasizes the potential for health promotion and disease prevention through the concepts of plasticity and phenotypic reversibility in response to positive environmental conditions. This body of research opens lines of intervention at the social level that can be reflected at the molecular level, thereby mitigating stress response (Franklin et al. 2012). This article aims to analyse scientific literature on epigenetics, with a focus on DNA methylation, in relation to individual and social protective/positive factors, such as resilience, self‐esteem, emotional regulation, social support and social and emotional functioning.

## 2 Methods

This scoping review follows the PRISMA Extension for Scoping Reviews (PRISMA‐ScR) (Tricco et al. 2018) guidelines and is registered in the Open Science Framework—OSF (osf.io/x9rjh). The review followed the phases: (1) establishment of the research question; (2) search for relevant studies; (3) selection based on the established inclusion criteria; (4) data analysis; and (5) communication of results.

The search was conducted across the Web of Science, Pubmed and Embase databases, targeting articles published online, with no restrictions on publication date or language. The first search was conducted in October 2018, and the second in May 2020, covering publications from 2008 to 2019. The descriptors and their synonyms in English were: (“*DNA methylation*”) *AND (resilience OR “emotional regulation” OR “self‐esteem” OR “social support” OR “emotional functioning” OR “social functioning”)*. The search was conducted by a librarian specialized in investigations on vulnerabilities in people who experience adversity and violence, with the support of authors. Two researchers independently evaluated the articles for inclusion; divergences were resolved through discussion with a third researcher.

The included studies have at their core DNA methylation in association with individual protective/positive factors (resilience, self‐esteem, emotional regulation, emotional functioning) or social protective/positive factors (social functioning, social support). Eligible studies included theoretical‐conceptual works, reviews or empirical research. Some articles that addressed factors such as maternal care/sensitivity, social interaction, social relationships and self‐control were included in the analysis. Additionally, two articles identified through the reference list were incorporated.

Publications were excluded if they did not meet the inclusion criteria of addressing DNA methylation and at least one protective/positive factor. Other exclusion criteria were studies focusing on DNA methylation related to pharmacological or toxicological therapies/analyses; research on plants; studies with exclusively biological approaches such as cellular behaviour; texts in books and abstracts published in scientific journals; full texts published in languages other than English, French, Portuguese or Spanish.

**FIGURE 1:**
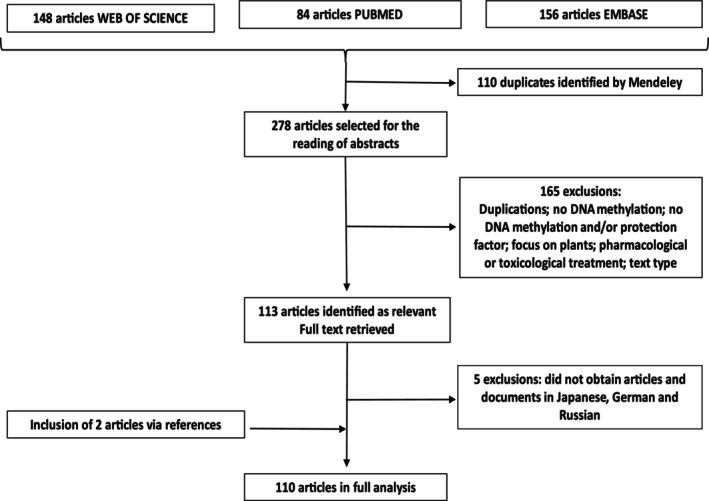
Stages of the literature review (from 2008 to 2019).

Figure 1 illustrates the process of collection, selection, inclusion and exclusion of the selected articles, which resulted in a final dataset of 110 full‐text articles included in the analysis. An Excel spreadsheet was created to organize data from the selected publications, which included the following categories: (a) *classification* of studies as empirical and theoretical/review studies, and their *profile*, including the age group investigated in the empirical articles (prenatal, childhood, adolescence, adults and elderly), year of publication; (b) study *focus* (human or animal); (c) *objectives*; (d) *epidemiological and epigenetic methods*, measurement tools/tests used and epigenetic approach including genes/CpGs investigated; (e) protective/positive factors; and (f) physical and mental health aspects addressed in the texts.

The data were analysed qualitatively and descriptively, following the categories mentioned above. Results were organized according to the definition and approach discriminated between resilience (*N* = 79 articles, Tables 1 and 2) and protective/positive factors (*N* = 31 articles, Tables 3 and 4). For clarity in presenting the findings, the articles were categorized into empirical studies (*N* = 55) and theoretical/review articles (*N* = 55). In publications where more than one protection/positive factor was addressed in the same study, we chose to prioritize in the tables the most relevant theme.

**TABLE 1:** Different meanings of resilience and epigenetics in empirical studies (*N* = 36).

Author/year	Human/animal	Sample characteristics	Genes	Theme/findings
**(1) Resilience to psychopathology, mental disorders, mental health** (*N* = 15)				
Chen et al. 2016	Human	12 participants with PTSD; 12 resilient participants to similar severity trauma exposure	591 genes	Increased gene expression may play an important role, acting differently between people with PTSD and resilient to similar severity trauma exposure. This expression may not be a consequence of DNA methylation.
Deng et al. 2017	Animal	361 male rats, 21 days old.	*BDNF*	Predictable chronic mild stress in adolescence decreased DNA methylation of the *BDNF* gene, promoting resilience to depression‐ and anxiety‐like behaviours in adulthood
Duclot and Kabbaj 2013	Animal	8 week‐old male rats	*BDNF*	The regulation of *BDNF* exon VI in the hippocampus as a critical regulator of stress resilience.
Elliott et al. 2010	Animal	Adult mice	*Crf* or *Crh*	Resilience to social stress coincides with functional DNA methylation of the *Crf* gene in adult mice.
Hammels et al. 2015	Animal	7 week‐old male and 30‐week‐old male retired breeder mice.	*DNMT3a*	It is observed increased density of Dnmt3a type II cells in the dentate gyrus of animals resilient to social defeat stress.
Ismaylova et al. 2017	Human	40 healthy adults	*SLC6A4*	Peripheral cells are more sensitive when studying *SLC6A4* promoter methylation and its associated risk for neural vulnerability and resilience for psychopathologies in which serotonin is implicated.
Kaufman, Wymbs, et al. 2018	Human	157 children (8–15 years old)	*OTX2*	Potential role for *OTX2* and *OTX2*‐related genes in the pathophysiology of stress‐related depressive disorders in children investigating risk and resilience in maltreated children.
Kim et al. 2017	Human	126 combat veterans with and 122 without PTSD	*BDNF*	Relevance of *BDNF* in modulating resilience and vulnerability to stress; association between higher DNA methylation of the *BDNF* promoter and PTSD diagnosis in combat‐exposed individuals.
Labonté et al. 2019	Animal	Male 7‐ to 12‐week‐old mice and 4–6 month old retired breeders	*Gadd45b*, *BDNF*	Contribution of *Gadd45b* and changes in DNA methylation in mediating the effects of social stress in the mesolimbic dopamine circuit. *Gadd45b* mRNA levels are increased in susceptible but not resilient mice.
Mehta et al. 2017	Human	96 male war veterans, 115 males from a general population as controls	*DOCK2* and others	Candidate genes and discovery of new genes associated with PTSD contribute to understanding the biological underpinnings of PTSD. Higher resilience scores are found in people without PTSD.
Mehta et al. 2018	Human	96 male war veterans, 115 males from a general population as controls	Accelerated DNAm age	DNAm age acceleration was significantly associated with resilience scores in veterans with PTSD; this association was likely driven by ‘self‐efficacy’, suggesting that among individuals already suffering from PTSD, some aspects of increased resilience may have a biological cost.
Rusiecki et al. 2012	Human	75 military with PTSD; and 75 with no PTSD diagnosis.	*LINE‐1*, *Alu*	Patterns of hypermethylation of *LINE‐1* in controls and of *Alu* in cases may suggest resilience or vulnerability factors.
Rutten et al. 2018	Human	Two cohorts: 93 male military servicemen; 98 male marines	*ZFP57*, *RNF39* and others	Three novel genomic regions where longitudinal decreases in DNA methylation across the period of exposure to combat trauma marks susceptibility for PTSD. Opposite effects in susceptibility and resilience to the effects of traumatic stress exposure were identified.
Sipahi et al. 2014	Animal Human	Publicly available genomes of several species	203 CpGs	PTSD and trauma resilience are associated with differential DNA methylation genome wide
Uddin et al. 2011	Human	23 individuals with and 77 without lifetime PTSD	*MAN2C1*	Investigating genes with epigenetic signatures indicative of increased risk for, or resilience to, PTSD, it was found that *MAN2C1* methylation levels modify cumulative traumatic burden on risk of PTSD.
**(2) Parent's mental health, maternal care, prenatal stress and neurobiological development** (*N* = 3)				
Ciernia et al. 2018	Animal	5 male and 5 female pups	*Gpr151*, *Slc5a7*, *Oxt* and others	Maternal care and stress resilience.
Mattern et al. 2019	Animal	Primiparous pregnant dams of wild‐type mice	*Bdnf*, *Fkbp5*, *Nr3c1* and others	Widespread DNA methylation changes due to prenatal enrichment, affecting all studied genes, 5 of 6 brain regions and both sexes.
Serpeloni et al. 2019	Human	122 grandmothers, 122 mothers and 120 children	*FKBP5*, *Nr3c1*	Following prenatal intimate partner violence, genomic sites in genes encoding the glucocorticoid receptor (*NR3C1*) and its repressor *FKBP5* were differentially methylated; more DNA methylation in heterochromatin‐like regions, associated with stress/disease resilience.
**(3) Resilience related to early life stress** (*N* = 7)				
Blaze 2018	Animal Human	Mice and 6 humans	*N*/A	Enduring molecular memory of stress and the possible mechanism by which select bioactive polyphenols may promote resiliency to stress.
Cramer et al. 2018	Animal	Male chicks	Various	An epigenetic mechanism involving histone modification and DNA methylation in a regulatory segment of *CRH* is involved in determining a resilient response to stress.
Duman and Canli 2015	Human	105 Caucasian males aged 18 to 77 (M = 28.51, SD = 13.82)	*5‐HTTLPR*, *SLC6A4*	Both early and recent life stress alter DNA methylation as a function of *5‐HTTLPR* genotype. Some of these changes are reflected in gene expression and cortisol response, differentially affecting individuals' stress response in a manner that may confer susceptibility or resilience for psychopathology upon experiencing stressful life events
Kaufman, Montalvo‐Ortiz, et al. 2018	Human	Two cohorts (160 and 74 children, 8‐ to 15‐year‐old)	*OTX2*	10 methylation sites were found to interact with adverse childhood experiences to predict body mass index; 6 sites exert a main effect in predicting body mass index. The investigation examines risk and resilience and psychiatric outcomes in maltreated children.
Pan‐Vazquez et al. 2015	Animal	33 mice (8 weeks old)	*NR3C1*	Exercise exerts a positive impact on stress resilience that could be mediated by decreasing *miR‐124* and increasing *Nr3c1* expression in the hippocampus.
Taff et al. 2019	Animal	3 swallows	Various	Individuals with brighter breast plumage and higher stress resilience had lower methylation at differentially methylated regions across the genome.
Theilmann et al. 1642	Animal	Male Wistar rats	*p11*	Behavioural differences from different vendors in resilience to chronic mild stress are reflected in epigenetic regulation and expression of *p11*
**(4) Biological age and development** (*N* = 2)				
Connolly et al. 2018	Human	453 White, middle‐aged, trauma‐exposed male and female veterans and civilians.	Accelerated DNAm age	Reflects on possible relationships between psychological resilience and telomere length. No evidence of an association between age‐adjusted telomere length and PTSD.
Miller et al. 2015	Human	292 African American teenagers, 17 to 20 years old	Accelerated DNAm age	Self‐control forecasts better psychosocial outcomes but faster epigenetic ageing in low‐SES youth; low‐SES youth, for whom resilience is a ‘skin‐deep’ phenomenon
**(5) Clinical and physiological conditions** (*N* = 6)				
Beach et al. 2018	Human	399 youth, mean age 11.7 years and 20.5 years in two assessments.	*SLC6A4*, *OXTR*	Reflects on biological mechanisms that potentially influence long‐term effects on resilience in young adulthood. For susceptible youth, preventive intervention may ‘get under the skin’, in a manner potentially beneficial for long‐term outcomes.
Kisliouk et al. 2017	Animal	Male chicks on the first day of life	*HSP70*	Depending on its stringency, exposure to heat in early life leads to either resilience or vulnerability to heat stress later in life. Heat‐Shock Protein Promoter HSP70 Exhibits Epigenetic Memory for Heat Stress.
Bove et al. 2018	Human	426 women prospectively followed until death	*OXPHOS* genes	Human females have a unique duration of postreproductive longevity, during which sex‐specific mechanisms influence later‐life mechanisms of neuronal resilience and vulnerability. The reproductive period and epigenetic modifications of the oxidative phosphorylation pathway are analysed.
Tyagi et al. 2015	Animal	Female Sprague–Dawley rats on 3rd day of pregnancy	*BDNF*	Diet can build an ‘epigenetic memory’ during brain formation that confers resilience to metabolic perturbations occurring in adulthood.
Wang et al. 2018	Animal	Male CD45.2 + C57BL/6 mice; CD45.1 + C57BL/6 mice as donors	*IL‐6*	Epigenetic modulation of inflammation and synaptic plasticity promotes resilience against stress.
McGuinness et al. 2018	Human	55 paired preperfusion and postperfusion renal biopsies (deceased donors)	Various	Physiological function and resilience to early life stress, biological age and adverse post‐transplant outcomes.
**(6) Resilience related to environmental/socioeconomic factors** (*N* = 1)				
Sevane et al. 2018	Animal	5 new and old‐world cattle breeds	*IRF6*, *GBX2* and others	Methylation patterns driven by profound environmental change provide a valuable pointer for the identification of biomarkers of resilience for improved performance and welfare.

**TABLE 2:** Different meanings of resilience and epigenetics in theoretical or review studies (*N* = 45).

Author, year	Genes	Theme/findings
**(1) Resilience to psychopathology, mental disorders, mental health** (*N* = 24)		
Binder and Holsboer 2012	*GR*, *FKBP5*	Low Cortisol and Risk and Resilience to Stress‐Related Psychiatric Disorders
Binder 2017	*FKBP5*	Interaction *FKBP5* and early adversity could contribute to stress‐related psychiatric disorders via a combined genetic and epigenetic disinhibition of *FKBP5* transcription. Analyses how adverse life events interact with genetic predisposition on the molecular level to shape risk and resilience to psychiatric disorders.
Boivin et al. 2015	N/A	Epigenetic influences shape neurodevelopmental risk and resilience
Cadet 2016	*DNMT3A*, *DNMT3B*, *DNMT1* and others	Epigenetic of stress, addiction and resilience. Psychobiology of resilience and alterations in epigenetic markers that have been observed in models of resilience.
Canli 2019	N/A	It hypothesizes that individual differences in vulnerability or resilience result from the balance of activated human endogenous retroviruses—HERVs with pathogenic versus protective functions in the brain. Epigenetic signals can be activated by stressful or traumatic experiences.
Cirulli 2017	*BDNF/NR3C1*	Much remains to be done to fully understand the effects of acute and chronic adverse conditions on the developing brain and about how pathology or resilience in the face of adversity is achieved.
Del Campo et al. 2018	*NR3C1*, *CRF*, *AVP*, *FKBP5* and others	Epigenetic programming of synthesis, release and/or receptor expression of common mediators participating in the risk/resilience for comorbid stress‐related disorders and coronary artery disease
Duclot and Kabbaj 2015	*BDNF*	Epigenetic mechanisms regulating the expression of BDNF and promoting vulnerability or resilience to the development of depressive‐like symptoms.
Dudley et al. 2011	*GR* and others	Epigenetic mechanisms mediating vulnerability and resilience to psychiatric disorders, including many that are stress‐related.
Griffiths and Hunter 2014	*DNMT1*, *DNMT3a*, *FKBP5* and others	Exposure to stress has both acute and lasting effects on epigenetic marks. Stress can be a positive response that keeps the individual alive and can increase resilience to future stressors.
Högfelt et al. 2018	*GAL*	Galanin may participate in the mechanism underlying resilience against a serious and major depressive disorder.
Howie 2019	*FKBP5* and others	Epigenetic regulation may be involved in resilience for posttraumatic stress disorder in the aftermath of trauma.
Jessen and Auger 2011	*DNMT*s and others	Epigenetic processes may confer sexually dimorphic resilience for developing a neurological or mental health disorder.
Lucero 2018	*NR3C1*, *SLC6A4* and others	An understanding of the way in which ecological systems shape trauma transmission, resilience and healing.
Murgatroyd and Spengler 2012	*DNMT1*, *DNMT3a* and others	Genetic variation in the epigenetic machinery and mental health. Insights into the molecular basis of vulnerability and resilience and advance tailored therapies.
Pfeiffer et al. 2018	*FKBP5*, *NR3C1*, *IL‐1*, *IL‐6*, *IL‐10* and others	Traumatic stress epigenetics and the transgenerational effects of prenatal stress exposures in humans. Aim to understand risk vs. resilience in the context of HPA‐axis genes.
Rutten et al. 2013	*5HTT*, *FKBP5* and others	Improving the understanding of the links between genetic endowment, environmental impact and gene–environment interactions with developmental psychology and biology is crucial for elucidating the neurobiological and psychological underpinnings of resilience
Schiele and Domschke 2018	*MAOA*, *COMT* and others	Epigenetics at the crossroads between genes, environment and resilience in anxiety disorders.
Sharma 2019	*CRFR1*, *FKBP5* and others	Twin studies and genetics‐based heritability analyses demonstrate that up to 40 to 50% of the variance in predicting PTSD following trauma is heritable. The mechanisms that mediate risk vs. resilience for PTSD following trauma exposure has yet to be elucidated.
Uddin et al. 2013	*SLC6A4*, *FOSB*, *DNMT1* and others	Investigating sex differences in DNAm among genes known to influence brain development may help to shed light on the sexually dimorphic risk for, or resilience to, developing PTSD and depression.
Walters and Kosten 2019	*DAT*, *5‐HTT*, *SlcA4*, *DNMTs* and others	Neurobiology of alcohol and substance use disorders, aiming insights into factors that support resiliency.
Zannas, Provençal, and Binder 2015	*SLC6A4*, *MAN2C1*, *COMT*, *FKBP5* and others	Examining the environmental, genetic and epigenetic factors that promote resilience to trauma/PTSD
Zannas and West 2013	*FKBP5*, *SLC6A4* and others	Epigenetics and the regulation of stress vulnerability and resilience
Zovkik et al. 2013	*BDNF*, *FKBP5*, *SLC6A4* and others	Epigenetic approaches in identifying markers of resilience that can be utilized to promote early intervention and develop therapeutic strategies to combat PTSD after symptom onset
**(2) Resilience related to parents' mental health, maternal care, prenatal stress and neurobiological development** (*N* = 6)		
Babenko et al. 2015	*DNMTs*, *GR* and others	Epigenetic mechanisms including changes in the level of noncoding RNAs, DNA methylation and histone modifications may potentially mediate stress resilience and stress vulnerability. Addresses stress‐induced perinatal and transgenerational epigenetic programming of brain development and mental health.
Champagne 2013	*Nr3c1*, *GR* and others	Glucocorticoid receptors, plasticity, resilience and the transmission of traits across generations
Champagne and Curley 2008	*Erα*	The methylation status of ER*α* has implications for reproductive behaviour, cancer susceptibility and recovery from ischemic injury, suggesting an epigenetic basis for risk and resilience across the life span.
Jawaid and Mansuy 2019	*FKBP5*, *BDNF*	Parental environmental enrichment has been associated with improved cognition and stress resilience in the offspring. Analyses intergenerational and transgenerational inheritance of behavioural phenotypes.
Papadopoulou et al. 2019	*NR3C1*, *FKBP5* and others	Preconceptual and in utero stressors form the foetal epigenetic profile together with the individual genetic profile, providing the background for individual stress response, vulnerability or resilience.
Tunc‐Ozcan et al. 2014	*Dio3* and others	Genetic and epigenetic risk factors interact to modulate vulnerability and resilience to foetal alcohol spectrum disorder
**(3) Resilience related to early life stress** (*N* = 5)		
Burns et al. 2017	*DNMTs*, *MECP2* and others	Plasticity of the epigenome during early‐life stress. Stress exposure is not deterministic and can have contradictory effects on adult outcomes, in some cases even being associated with increased resilience to later stressors.
Chocyk et al. 2013	*11β‐HSD2*	Resilience to early‐life stress related to anxiety and mood disorders depend on the interaction between individual genetic predispositions, early‐life experiences and later‐life environment.
Ehlert 2013	*FKBP5*, *BDNF* and others	The continuum of trauma‐provoked aftermath reaches from healthy adaptation with high resilience, to severe maladjustment with co‐occurring psychiatric and physical pathologies in children, adolescents and adults
Franklin et al. 2012	*FosB*, *5HT1AR* and others	Evidence implicating altered functions and connectivity of the neuroendocrine system and of hippocampal, cortical, reward and serotonergic circuits in the establishment and the maintenance of stress resilience
Jiang et al. 2019	*FKBP5*, *MAOA*, *NR3C1* and others	The heritability of adverse childhood experiences related to phenotypes (PTSD, resilience) is low to moderate and, moreover, is very variable for a given phenotype, which implies that gene by environment interactions (such as through epigenetic modifications) may be involved in the onset of these phenotypes.
**(4) Biological age and development** (*N* = 3)		
Barter and Foster 2018	*DNMTs BDNF* and others	Epigenetic–environmental link may contribute to the accumulation of cellular damage, susceptibility or resilience to stressors and variability in the trajectory of age‐related cognitive decline.
Folguera‐Blasco 2018	N/A	Cellular ageing, and its reversal, might result from stochastic translation of metabolic inputs into resilient/plastic cell states via epigenetic regulation systems.
Plomin and Simpson 2013	N/A	The future of genomics for developmentalists: genetic, epigenetic, risk and resilience.
**(5) Clinical and physiological conditions** (*N* = 2)		
Denk et al. 2014	*COMT*, *OPRM1*, *SLC6A4* and others	Mechanisms that underlie resilience toward developing chronic pain.
Stover et al. 2018	PGC‐1a, PPAR‐delta	Revealing diet–epigenetic relationships has the potential to transform nutrition science by increasing our fundamental understanding of the resilience of living organisms in responding to environmental perturbations.
**(6) Resilience related to environmental/socioeconomic factors** (*N* = 5)		
Ohm 2019	H3K4, 5‐HMC	Different types of environmental stressors affecting the epigenome, epigenetic reprogramming, altered allostatic load and resilience in African American women's health.
Nabeshima and Kim 2013	*BDNF*, *DISC1* and others	Epigenetic marks, environmental factors and depression. Possible. Resilience is heightened because of a favourable nurturing environment after birth or perhaps due to exercise.
Hertzman 2012	*GR*	Biological embedding, socioeconomic gradients, stress and resilience
Hertzman 2013	N/A	A gradient in developmental health suggests that the emergence of a multifaceted resilience early in life is the best place to look for evidence of biological embedding.
Tian and Marsit 2018	*FKBP5*, *NR3C1* and others	Due to innate resilience, epigenetic changes caused by environmental exposures may not always lead to impairment but may allow the organisms to achieve positive developmental outcomes through appropriate adaptation and a buffering response

**TABLE 3:** Emotional and social protective factors and epigenetics in empirical studies (*N* = 21).

Author, year	Human/animal	Sample characteristics	Genes	Protective factors	Theme/findings
**(1) Maternal psychosocial functioning, family, social functioning and interaction—diversity of genes** (*N* = 13)					
Alisch et al. 2014	Animal	23 young male monkeys with an average age of 1.3 (SD 0.2) years.	*BCL11A*, *JAG1*	Emotional functioning (anxious temperament)	Increased DNA methylation reduces *BCL11A* expression and contributes to the anxious temperament phenotype. Negative correlation between *JAG1* expression and anxious temperament severity. Roles in neurodevelopmental processes, including neurite arborization and the regulation of neurogenesis.
Booij et al. 2015	Human	29 women with anorexia nervosa, 15 normal‐weight control women.	*NR1H3*, *PXDNL*	Social functioning	The chronicity of anorexia nervosa was associated with gene pathways associated with anxiety and altered social functioning.
Chang et al. 2016	Animal	Pregnant rats: 12 in control and 13 in bisphenol A groups.	*Erα*	Learning/memory and anxiety‐like behaviours (emotional regulation)	Perinatal exposure to bisphenol A impairs learning/memory function and elevated DNA methylation of the ERα gene in the hippocampus may be involved. No substantial change in anxiety‐like behaviours was observed.
Chen et al. 2012	Human	140 individuals: patients with schizophrenia, healthy biological siblings, healthy subjects without a family history of mental disorders.	*MB‐COMT*	Psychosocial functioning	Siblings of patients with schizophrenia have significant deficits in cognition and psychosocial functioning, which may not be associated with *MB‐COMT* methylation in peripheral leukocytes.
Conradt et al. 2016	Human	128 dyads—mothers (mean age 30.5 years) and infants (average age 19.1 weeks).	*NR3C1*, *11*β*‐HSD2*	Maternal sensitivity and responsiveness, social buffering	Mothers with depressive symptoms who were more responsive and who engaged in more appropriate touch during face‐to‐face play had infants with less DNA methylation of *NR3C1* and *11‐HSD2* compared with mothers with depressive symptoms who were also insensitive.
Di Sante et al. 2018	Human	31 one adults (24 Females, 27 Males; M = 36 years, SD = 1.2) from two longitudinal cohorts. Individuals were included in the cohort at age 6.	*FKBP5‐intron‐7 and NR3C1‐1 F‐promoter*	Emotional regulation, resilience	Two‐year stability of *NR3C1‐1* and *FKBP5* methylation. NR3C1 down‐regulation may be associated with greater frontal‐limbic connectivity in healthy adults and may underline more efficient neural and emotional regulation and, potentially, higher resilience to the potential adverse effects of such epigenetic phenotypes in healthy adults.
Folger et al., 2019	Human	53 at‐risk mother–child dyads from a longitudinal cohort study.	*NR3C1*	Social–Emotional functioning	Higher mean DNAm of the *NR3C1* exon 1F promoter was significantly associated with more positive infant social–emotional functioning
Fries et al. 2014	Human	24 patients with bipolar disorder, 18 siblings, 26 controls.	*FKBP5*	Coping, resilience	*FKBP5* mediates and epigenetically‐induce modulation of the stress axis in bipolar disorder patients. Stress resilience and coping mechanisms as key elements in the development and progressive course of bipolar disorder.
Kingston et al. 2019	Human	285 participants.	*DRD2*, *DAT1*, *COMT* and others	Parents' mental health, resilience, social support (environment, socioeconomic status)	Physiologically, lack of resilience is most evident in changes to biomarkers related to the stress response, including immune function and metabolomics. Cortisol fluctuations have been associated with exposure to disaster and trauma in children, and it was found association of genetic modification to child resilience.
Lapp et al. 2019	Human	90 Participants (48.4% female, mean age = 32.12 ± 15.10)	*NR3C1*, *FKBP5*, *NADH*	Social support, resilience	Multiple contributions across psychological, genetic, epigenetic and social domains to vulnerability and resilience in hypothalamic–pituitary–adrenal axis regulation.
Lee et al. 2014	Animal	48 adult female monkeys	*NR3C1*, *GR1F*	Coping, resilience	Stress inoculation enhances behavioural and hormonal aspects of coping without significantly influencing *GR1F* promoter DNA methylation as a mechanism for *NR3C1* transcription regulation.
Silva 2018	Animal	20 female Wistar rats (control group = 8 and fluoxetine group = 12) during pregnancy	Various.	Social interaction	An increase in the global DNA methylation profile of hippocampus was associated with the early exposure to fluoxetine, which in turn was associated with a reduction in the social interaction time and to a decrease in the plasma corticosterone level when animals were submitted to restraint stress. Maternal exposure to fluoxetine during gestation and lactation results in a long‐lasting impact on the DNA methylation of hippocampus, affecting the social behaviour.
Surkan et al. 2019	Human	250 African‐American mothers from a birth cohort with an epigenetic study on preterm birth.	*PRDM16*, *BANKL* and others	Social support	Self‐reported maternal psychosocial lifetime stress and stress during pregnancy was not associated with DNAm alterations. Absence of support from the baby's father was significantly associated with maternal DNAm changes in some genes. Lack of support from family and friends was associated with maternal DNA hypermethylation on multiple genes.
**(2) Social interaction and perception, maternal involvement, emotion processing, attachment, attention—the role of oxytocin** (*N* = 8)					
Chen et al. 2019	Human	304 healthy male and female subjects, randomized to treatment with either intranasal oxytocin, placebo or intranasal vasopressin.	*OXTR*	Social functioning; social interactions	*OXTR* methylation status may influence Oxytocin effects on mentalizing, attention and reward processing during social interactions (brain regions: the precuneus and visual cortex). OXTR methylation may be important to consider if exogenous Oxytocin is used to treat social behavioural disorders in the future.
Ebner et al. 2019	Human	Young and older adults.	*OXTR*	Social functioning, attachment	Lower levels of *OXTR* methylation and higher plasma oxytocin levels were associated with less self‐reported attachment anxiety in young but not older participants. Lower levels of *OXTR* methylation were associated with higher self‐reported attachment avoidance.
Kogan et al. 2018	Human	358 rural young African American men (age 19 at baseline)	*OXTR*	Emotional functioning, supportive and prosocial bonds	Early adversity was associated with *OXTR* methylation indirectly via contemporary prosocial relationships. OXTR methylation was a proximal predictor of changes in substance‐related symptoms. No evidence for a direct association of self‐reported childhood trauma with *OXTR* methylation status.
Krol, Puglia, et al. 2019	Human	101 human mother‐infant dyads	*OXTR*	Social support, maternal engagement	Oxytocin receptor gene is dynamic in infancy and its change is predicted by maternal engagement and reflective behavioural temperament.
Krol, Moulder, et al. 2019	Human	7‐month‐old infants (*N* = 84)	*OXTR*	Social interactions, emotion processing/regulation.	Interaction of *OXTR* methylation and facial emotion. Higher levels of *OXTR* methylation, and a presumably reduced ability to use endogenous oxytocin, were associated with enhanced responses to anger and fear and attenuated responses to happiness.
Milaniak et al. 2017	Human	91 youth exposed to pre‐ and postnatal adversity with established conduct problem trajectories.	*OXTR*	Social cognition, prosocial behaviour, resilience	*OXTR* methylation at birth is related to resilience in the conduct problems domain in middle childhood. Youth who were resilient in terms of conduct problems also had lower emotional problems, higher prosocial behaviour and better social cognition as compared with youth who were non resilient in terms of conduct problems.
Puglia et al. 2018	Human	54 neurotypical Caucasians of European descent (31 males) aged 18–30 (M = 21.2, SD = 3.0) years	*OXTR*	Social attention	Epigenetic modification to the oxytocin receptor is associated with increased neural response and decreased functional coupling between regions of the salience and attentional control networks during selective social attention.
Puglia et al. 2015	Human	98 healthy adults	*OXTR*	Social perception and emotion processing	High levels of *OXTR* methylation were associated with greater amounts of activity in regions associated with face/emotion processing and with decreased functional coupling of amygdala with regions involved in affect appraisal and emotion regulation.

**TABLE 4:** Emotional and social protective factors and epigenetics in theoretical or review studies (*N* = 10).

Author, year	Genes	Protective factors	Theme/findings
**(1) Maternal psychosocial functioning, family and social functioning and interaction—diversity of genes** (*N* = 8)			
Díaz‐Anzaldúa et al. 2011	*MAOA*, *COMT* and others	Social competence, self‐esteem, resilience	Childhood maltreatment, including neglect as well as physical and sexual abuse, is associated with developmental difficulties, low social competence and self‐esteem, and it is an important risk factor for binge drinking in adolescence and alcohol dependence in adulthood. Childhood maltreatment may interact with factors such as variants of the monoamine oxidase‐A and catechol‐omethyltransferase gene; Met158 variant in the *COMT* gene may confer risk and resilience to alcohol dependence in different environments.
Gemmel et al. 2018	*FKBP5*	Social behaviours, interactions	Perinatal selective serotonin reuptake inhibitor medication—SSRI affect maternal care and neurodevelopmental outcomes related to social affiliative behaviours in offspring, such as play behaviours, social interactions, reproductive behaviours and maternal care of the next generation. Early life exposure to SSRIs can alter related neurobiology and the epigenome.
Holmes et al. 2019	*CTRA*, *NR3C1* and others	Spiritual support system	On psychosocial and immune cell response and gene expression, current data on human models do implicate appropriate gene expression via the *CTRA* and *NR3C1* gene in the spiritual network support system implicated in malignant neoplasm remission.
Noro and Gon 2015	*Gaba*, *BDNF*, *GR* and others	Maternal Care	Post‐natal maternal care is found to cause effects on the hypothalamic–pituitary–adrenal (HPA) axis activity participating in the building of stress vulnerability and resilience.
Roth 2013	*GR*, *GABA*, *GAD1* and others	Maternal care, resilience	Maternal care also promotes epigenetic changes of additional genes and epicentres of stress regulation, cognitive control, addiction and maternal behaviour. DNA methylation has emerged as a leading candidate biological pathway linking gene–environment interactions to long‐term and even multigenerational trajectories in behavioural development, including the vulnerability and resilience to psychopathology.
Roth and David Sweatt 2011	*GR*, *Erα* and others	Maternal care, resilience	Epigenetic mechanisms and environmental shaping of the brain (maternal care) during sensitive periods of development. Experiences during sensitive periods of development influence DNA methylation patterns of several genes. These experience‐induced DNA methylation patterns represent stable epigenetic modifications that alter gene transcription throughout the lifespan and promote specific behavioural outcomes.
Shields 2017	*NR3C1*, *FKBP5* and others	Social support, resilience	Little research to date has focused on positive mediating influences such as social support, coping and spirituality that might offset the adverse effects of stress or adverse life circumstances and promote resilience and health.
Wheeler 2019	N/A	Social interactions	The importance of the epigenome for social‐related neural circuits
**(2) Social interaction and perception, maternal involvement, emotion processing, attachment, attention—the role of oxytocin** (*N* = 2)			
Holt‐Lunstad 2019	*OXTR*	Social bonding	*OXTR* has also been implicated in other forms of social bonding such as pair bonding and familial ties, and social processes such as social cognition and trust, which apply to a variety of social relationships and roles
Maud et al. 2018	*OXTR*	Emotional functioning	Increased OXTR DNAm in general impairments in social, cognitive and emotional functioning. Decreased OXTR DNAm in specific patterns of impairment related to mood and anxiety disorders (but not in all).

## 3 Results

### 3.1 Descriptive and Methodological Aspects of the Selected Publications

Among the 110 articles analysed, 11.8% were produced between 2008 and 2012, 41.8% between 2013 and 2017 and 46.4% between 2018 and 2019, indicating a significant and gradual increase over time. Publications were predominantly featured in journals focusing on neuroscience and the human brain, psychiatry and psychopathology, with fewer publications in the public health field (Beach et al. 2018; Walters and Kosten 2019; Tian and Marsit 2018). The distribution of empirical and theoretical/review studies was relatively balanced. Among the empirical studies, epidemiological research involving human participants predominates, including surveys, case–control, cohort and clinical studies, followed by experimental studies using animal models; two studies employed postmortem mathematical and biological methods. Of the 55 empirical studies, most are based on investigations with humans (62.9%), especially adults, followed by studies with nonhuman animal models (37.1%), such as monkeys, rats, mice, chickens and swallows.

### 3.2 Epigenetic Approaches

A total of 55 empirical studies analysed epigenetic mechanisms (DNA methylation), with 63.0% employing a candidate gene approach and 37.0% addressing the genome‐wide methylation profiles. Three studies evaluated accelerated epigenetic ageing (Walters and Kosten 2019; Miller et al. 2015; Mehta et al. 2018).

Studies exclusively involving human participants (*N* = 34) evaluated DNA methylation of 5040 individuals (42.9% female), spanning from infants to the elderly, with a mean age of 27.4 years reported in 28 studies. Peripheral blood and saliva were the primary DNA sources in human studies, while brain tissue biopsies were commonly used in animal studies (*N* = 21). Different methodologies to detect methylated CpG islands were used in both candidate gene and genome‐wide approaches.

Beyond DNA methylation, additional epigenetic mechanisms such as histone modification (31 studies) and noncoding RNA, primarily miRNA (15 studies), were also identified, indicating that these alternative mechanisms were especially prominent in resilience‐related studies, as well as in theoretical and review studies.

The candidate gene studies investigated various genes, including *OXTR*, *NR3C1*, *SLC6A4*, *BDNF*, *FKBP5*, transposable elements *Alu* and LINE‐1, *CRH*, *OXPHOS*, Dnmt3a, *OTX2*, *HSP70*, *POU2F1*, *MB‐COMT*, *5‐HTTLPR*, *HSD11B2*, *E2F*, *MT‐ND6*, *Htr1a*, *MAOA*, *NOS1*, *Gadd45b*, *MYC*, *MTORC1*, *Crf*, *ERα* and *p11 gene*. Genome‐wide and review studies addressed a broader range detailed in Tables 1, 2, 3, 4, which present the prioritized genes in the publications, organized by study type, resilience and protective/positive factors.

### 3.3 Resilience as a Diverse Set of Concepts

Resilience was addressed in 71.8% of the articles, primarily defined as the capacity to overcome adversity as an innate human attribute, characterized by: (1) ability to resist, adapt, recover, overcome; and (2) ability to overcome stress, adversity and trauma (Beach et al. 2018; Walters and Kosten 2019; Tian and Marsit 2018; Connolly et al. 2018; Miller et al. 2015; Mehta et al. 2018; Babenko et al. 2015; Kingston et al. 2019). Most studies conceptualized resilience as a positive adjective of success in the face of adversity, the absence of disease or injury (Dudley et al. 2011; Binder and Holsboer 2012). Others prioritize the individual's performance (physical and emotional attributes), considering traits and behaviours such as personal motivation and drive, work ethics, persistence and willingness to engage in challenging and demanding tasks, mediated by a positive personality style, cognitions and coping behaviours (Connolly et al. 2018).

However, the conceptualization of resilience as an individual capacity encompasses attributes beyond the physical and emotional spheres, including the biological and physiological basis to maintain allostasis and respond effectively to acute and chronic threats to survival (Ohm 2019). From the perspective of public health, Cadet (2016) emphasizes the relevance of interactions between family characteristics, community involvement and genetic markers, allowing the environmental regulation of physiological and behavioural systems and, therefore, a general adaptive response.

At the biological and physiological level, the reactivity of the hypothalamic–pituitary–adrenal (HPA) axis in response to stress stands out as important for the understanding of resilience: increased expression of glucocorticoid receptors (GR*)* in the hippocampus, correlated with greater resilience to stress; genetic and epigenetic variations; various systems such as mesolimbic dopaminergic neurons, noradrenergic neurons, *BDNF* and neuropeptides such as opioids and corticotropin‐releasing hormone (CRH), among others; polymorphism of genes encoding dopamine receptors and the serotonin transporter (Babenko et al. 2015; Connolly et al. 2018; Cadet 2016; Högfelt et al. 2018; Theilmann et al. 1642). Among the anatomical brain structures involved in stress responses are subcellular structures such as mitochondria and telomere length, which are related to reactive oxygen species (ROS), inflammation and intracellular calcium levels in myocytes and neural cells. Additionally, chemical mediators, particularly neuroimmune hormones, play a key role. The interrelationship between the nervous and cardiovascular systems in the management of stress is highlighted (Del Campo et al. 2018).

In relation to human behaviour, the way to understand adverse situations is highlighted. According to Franklin et al. (2012), resilient individuals would perceive adversity as less threatening and thus develop adaptive physiological and psychological responses, as well as a way to deal with problems, the so‐called coping strategies (Binder and Holsboer 2012; Binder 2017). Regarding the influence of the environment on the formation of resilience, the following factors stand out: maternal care (Ciernia et al. 2018; Nabeshima and Kim 2013), social environment where people live (Hertzman 2012, 2013), situations of social stress (Hammels et al. 2015) and heat in animals (Kisliouk et al. 2017). Resilience is a dynamic process and can be actively invoked by molecular, hormonal, neural and behavioural mechanisms that neutralize existing vulnerabilities or prevent the expression of vulnerable phenotypes (Lucero 2018; Milaniak et al. 2017; Zannas and West 2013).

Some types of resilience are named as resilience to stress (Burns et al. 2017; Babenko et al. 2015; Binder and Holsboer 2012; Binder 2017; Barter and Foster 2018); neurological or neural resilience (Franklin et al. 2012; Bove et al. 2018; Tyagi et al. 2015); psychological resilience (Connolly et al. 2018); resilience to social stress and the environment (Binder 2017; Wang et al. 2018; Elliott et al. 2010); resilience to psychopathologies as depression‐ and anxiety‐like behaviours (Deng et al. 2017) and post‐traumatic stress disorder (PTSD) (Chen et al. 2016); mental illness/health (Connolly et al. 2018; Chen et al. 2016); and epigenetic resilience (Tian and Marsit 2018).

### 3.4 Resilience and Epigenetics

Tables 1 and 2 summarize the findings from 79 articles that address resilience in its different conceptualizations. The empirical studies (*N* = 34; Table 1) investigated diverse samples and topics, across human and animal models. Many theoretical/review studies (*N* = 45; Table 2) emphasized stress regulation, brain development and mental health. The studies on both tables were categorized according to the primary focus of the article and are presented in the analytical themes below.

1. **Focus on psychopathology, mental disorders and mental health** are predominant in Tables 1 and 2 (*N* = 39). Studies focused on PTSD (Sipahi et al. 2014; Rutten et al. 2018), mood disorders (alone and in comorbidity with obesity) and affective disorders (Cadet 2016); neurodevelopmental disorder and disability (Jessen and Auger 2011; Boivin et al. 2015); attention deficit and hyperactivity disorder and Rett syndrome (Uddin et al. 2011); and suicide (Dudley et al. 2011; Binder and Holsboer 2012; Binder 2017; Schiele and Domschke 2018). Stress‐related disorders (Del Campo et al. 2018) are further investigated.
  Some of the genes pointed out in the articles on psychopathology are: epigenetic alterations in *LINE‐1*, *MAN2C1* and *BDNF* in PTSD (Kim et al. 2017; Rusiecki et al. 2012; Uddin et al. 2013); *DNMT3a* appears associated with brain development and plasticity and social behaviour (Hammels et al. 2015; Jessen and Auger 2011); CRH is involved in determining resilient stress response (Elliott et al. 2010); and *Gadd45b* is associated with changes in DNA methylation in situations of social stress (Labonté et al. 2019).
  While most articles on this topic prioritized the notion of resilience as the absence of disease, two texts differed because they measured resilience using quantitative scales. Mehta et al. (2017) observed differential DNA methylation of 17 candidate genes associated with the severity of PTSD symptoms spanning *BRSK1*, *LCN8*, *NFG* and *DOCK2*, with lower resilience scale scores among people with the disorder. Mehta et al. (2018) found an increase in the acceleration of epigenetic age associated with increased resilience among veterans with PTSD, only in the self‐efficacy factor. Among controls without PTSD, increased resilience scores were associated with decreased acceleration of epigenetic age in veterans and the civilian population.
2. **Parent's mental health, maternal care, prenatal stress and neurobiological development** (*N* = 9). Studies on violence in pregnancy (Serpeloni et al. 2019), foetal alcohol spectrum disorder (Tunc‐Ozcan et al. 2014), memory problems (Ciernia et al. 2018) and behaviour problems (Jawaid and Mansuy 2019) stand out.
  Maternal care is considered essential to positively impact brain development, reducing anxiety and allowing resilience to behaviours such as depression, as well as improving learning and memory (Ciernia et al. 2018), as it can alter GR levels, suggesting a neurobiological basis for attenuated stress reactivity (Papadopoulou et al. 2019). Maternal care in the postnatal period affects neurodevelopment related to playful behaviours, social interactions, memory, reproductive behaviours and maternal care of the next generation, promoting resilience to stress throughout life with a modest but significant decrease in overall DNA methylation (Ciernia et al. 2018). Jawaid and Mansuy (Jawaid and Mansuy 2019) reveal that children and grandchildren of genocide survivors exhibit increased emotional disturbances; and Champagne (Champagne 2013) points out that interactions between gene and environment reproduce within and through a family's generations.
  Serpeloni et al. (2019) revisit the hypothesis that there is a protective effect of prenatal stress, since the presence of methylation suggests that children exposed to prenatal stress would have higher production of GR, resulting in increased negative feedback of the HPA axis and, therefore, greater resilience to stress. The emphasis on interpersonal relationships expressed in maternal care was conceived as an environmental event that can produce epigenetic changes related to the biological bases of vulnerability to stress (Ciernia et al. 2018).
  Different genes were associated with parents' mental health, maternal behaviours and maternal care, with key findings on genes *NR3C1* and its repressor *FKBP51* connected to intimate partner violence in prenatal care (Serpeloni et al. 2019; Papadopoulou et al. 2019). Ciernia et al. (2018) detected 9439 differentially methylated regions associated with increased maternal care, especially 20 main genes, among which are *Gpr151*, *Slc5a7*, *Slc6a4*, *Oxt* and others.
3. **Early life stress** (*N* = 12). There are stressful events that affect health, especially adverse experiences in childhood (child maltreatment, chronic stress, stress resilience in young animals). The relevance of the life cycle is highlighted, especially stress or adversities that occur in the first years of life, in the acquisition of resilience (Burns et al. 2017; Jiang et al. 2019). Cramer et al. (2018) reveal that early experiences have been shown to be beneficial for the development of resilience to stress. Ehlert (2013) reports altered function and interconnectivity of the neuroendocrine system and diverse brain circuits (hippocampal, cortical, reward and serotonergic) in establishing and maintaining resilience to stress. Studies identified epigenetic changes in genes like *FKBP5*, *BDNF* (Ehlert 2013); *SLC6A4* (Duman and Canli 2015) among others which, in addition to acting on the nervous system and the stress response, may be involved in aspects of the cardiovascular system, especially through the regulation of the hypothalamic–pituitary–adrenal axis and the inflammatory response.
4. **Biological Age and Development** (*N* = 5). Studies in Tables 1 and 2 highlight the importance of the interaction between epigenetics, reprogramming cellular identity (Folguera‐Blasco 2018), senescent physiology (Barter and Foster 2018; Connolly et al. 2018) and environmental factors influencing gene transcription and development, as done by Miller et al. (2015), who advised that young people from lower social stratum would have accelerated epigenetic ageing.
5. **Clinical and Physiological Conditions** (*N* = 8). Studies addressed clinical conditions such as ischemic lesion of the retina, reproductive system and neuroendocrine changes (Bove et al. 2018), chronic pain (Denk et al. 2014), skin diseases; nutrition (anorexia, obesity) (Stover et al. 2018), inflammatory processes (Wang et al. 2018), substance use (Beach et al. 2018), kidney disease and transplants (McGuinness et al. 2018). Epigenetic alterations were related to diet and metabolic perturbations (Tyagi et al. 2015; Stover et al. 2018) and heat stress (Kisliouk et al. 2017) in genes like *SCL6A4*, *OXTR*, *BDNF*, *IL‐6*, *COMT*, *FKBP5* and others.
6. **Resilience related to environmental/socioeconomic factors** (*N* = 6). Studies highlight different conditions associated with resilience, such as the involvement of environmental factors in development (Tian and Marsit 2018; Nabeshima and Kim 2013) and the socioeconomic gradient associated with a broad range of health outcomes (Hertzman 2012, 2013). The study with African American women identified high allostatic load and greater resilience to external factors (Ohm 2019). Major environmental changes such as climate change that had an impact on methylomes are related to immune response, nervous system, energy management, heat resistance and skin and coat attributes (Sevane et al. 2018). Some genes mentioned *are IRF6*, *GBX2*, *DISC1*, *BDNF*, *FKBP5*, *NR3C1* and others.

### 3.5 Protective/Positive Factors and Epigenetic

Tables 3 and 4 summarize the findings from 31 articles that address protective factors, particularly focusing on genes associated with oxytocin signalling, such as OXTR and OXT2, primarily through empirical studies. It was common to find the association between resilience and several protective/positive factors related to individual and social bonds and competencies, such as social/emotional functioning/regulation (Di Sante et al. 2018; Chen et al. 2012, 2019; Folger et al. 2019; Kogan et al. 2018; Alisch et al. 2014; Booij et al. 2015; Maud et al. 2018), social cognition (Milaniak et al. 2017), maternal care (Roth and David Sweatt 2011; Roth 2013; Conradt et al. 2016; Noro and Gon 2015), social behaviours/interaction (Krol, Moulder, et al. 2019; Gemmel et al. 2018; Silva 2018; Wheeler 2019; Holt‐Lunstad 2019), spiritual/social support (Kingston et al. 2019; Surkan et al. 2019; Krol, Puglia, et al. 2019; Lapp et al. 2019; Shields 2017; Holmes et al. 2019), attachment (Ebner et al. 2019), social attention/perception (Puglia et al. 2018, 2015), social competence and self‐esteem (Díaz‐Anzaldúa et al. 2011) and coping (Fries et al. 2014; Lee et al. 2014). Different from the studies mentioned in the previous section, most of the studies presented here use standardized instruments, clinical tests, drawing techniques, photographs, games and observations of interpersonal interaction to deepen the understanding of these protective/positive factors.

A wide range of instruments has been utilized to evaluate different constructs, including: emotional and social functioning (Chen et al. 2012; Folger et al., 2019); social adjustment (Chen et al. 2012); social support/assistance (Folger et al., 2019; Puglia et al. 2018); cognitive skills such as speech, attention, perception and memory (Chen et al. 2012; Puglia et al. 2018; Ebner et al. 2019); sensitivity, involvement and maternal engagement (Kogan et al. 2018; Conradt et al. 2016); parental social competence (Krol, Puglia, et al. 2019); child development and behaviour and expression assessment (Puglia et al. 2018); adult attachment (Ebner et al. 2019); personality inventory (Ebner et al. 2019); and social functioning (Chen et al. 2012).

Prominent topics include emotional and behavioural functioning (Alisch et al. 2014); coping strategies (Fries et al. 2014); *s*ocial affiliation behaviours (Gemmel et al. 2018); social interactions as opposed to low social status and stress (Lapp et al. 2019); maternal care (Di Sante et al. 2018; Roth 2013; Noro and Gon 2015); positive mediating influences (social support, coping and spirituality) (Shields 2017).

The reviewed articles on protective/positive factors do not primarily focus on psychiatric disorders (Tables 3 and 4). Instead, two major thematic groups emerged:

1. **Maternal psychosocial functioning, family and social functioning and interaction** (*N* = 21). Studies examine family and social functioning associated with gene diversity, with varied approaches, including attention, vigilance and memory of siblings of patients with schizophrenia (Chen et al. 2012); depression, as opposed to maternal sensitivity (Conradt et al. 2016); maternal social stress in pregnancy and social support received (Surkan et al. 2019); and social–emotional functioning of infants in adverse psychosocial environments (Folger et al., 2019). Some genes related are *NR3C1*, *TOR3A*, *IQCB1*, *C7orf36*, *MYH7B*, *PRDM16*, *BANKL* and *11‐HSD2*.
2. **Role of oxytocin related to social interaction and perception, maternal involvement, emotion processing, attachment and attention** (*N* = 10). Attributes such as positive social interactions, prosocial relationships, perception of facial emotions, maternal involvement, temperament, cognitive abilities, social and affective functioning, attachment and social information (Chen et al. 2019; Kogan et al. 2018; Puglia et al. 2015, 2018; Ebner et al. 2019) are central to this theme. *OXTR* gene was the main target of the studies for understanding the neuropeptide's influence on social perception, emotion processing and social‐affective functioning.

## 4 Discussion

The scoping review conducted in this study highlights a significant increase in scientific publications focusing on the relationship between epigenetics, particularly DNA methylation, resilience and protective/positive factors from 2008 to 2019. The primary findings indicate that resilience is the most extensively explored topic within this field, with a focus on its connection to mental health disorders, parental mental health, early life stress, biological development, clinical conditions and environmental/socioeconomic factors. A predominant reliance on candidate gene approaches, particularly concerning genes like *OXTR*, *FKBP5* and *NR3C1*, was observed, although genome‐wide methylation studies are gradually gaining traction.

The state of the art on DNA methylation, resilience and protective/positive factors reflects the growing interest in this field in recent years, underscoring the significance of both human and animal research. However, it also highlights that the field is still in its early stages, requiring further theoretical and methodological refinement, along with substantial financial resources to support comprehensive epigenetic analyses. The emerging knowledge about epigenetics, resilience and protective/positive factors has become a pivotal theme in neuroscience, significantly enhancing our understanding of human behaviour and mental health. This knowledge is essential for shaping future strategies and interventions aimed at improving the well‐being of future generations.

Several key aspects identified in the review warrant attention. First, the varied definitions of resilience complicate the comparison of studies. While many definitions equate resilience with the absence of disease, oversimplifying this complex concept, others advocate for a broader definition that encompasses both individual and collective dimensions, reflecting an evolution in discourse. The small number of articles that use resilience scales deserves to be highlighted, demanding more investment in measuring the construct. Additionally, the high frequency of epigenetic investigations in this area indicates important directions for future research. Another notable observation is the diversity of genes associated with resilience studies, which vary according to the broad outcomes investigated.

The large number of studies examining protective and/or positive factors, such as social and emotional functioning, maternal care and social interaction, highlights a growing research area with relevance to mental health. This indicates opportunities for promising research directions aimed at promoting healthier developmental trajectories.

DNA methylation in genes involved in the activity of the HPA axis is highlighted, as is the dopaminergic, noradrenergic and serotonergic systems and their association with the immune system (Mehta et al. 2020). Genomic‐scale analyses of DNA methylation profiles are crucial for establishing DNA signatures that assess protection or promote positive outcomes, uncover new regulatory mechanisms and provide a deeper understanding of the complex biological pathways that are susceptible to environmental changes (Serpeloni et al. 2016).

The high number of reviews is partly due to the introduction of epigenetics in health research in recent years, demanding better knowledge about connections with cardiovascular, immune, endocrine and central nervous systems. It also accompanies the growing demand for high‐quality scientific evidence, in which the role of systematic reviews in evidence‐based medicine stands out. Worldwide, in 2010, fourteen reviews were published per day, and in 2019, this number increased to 80 per day (Hoffmann et al. 2021). While many themes addressed in this review exhibit considerable repetition, the novelty of the subject and the challenges it presents may have prompted researchers to recognize the need for a theoretical review essential for guiding their future studies.

Another aspect highlighted in the review concerns the similarities and differences between human and animal studies. The same epigenetic mechanisms are often examined, with a high frequency of overlapping genes and shared environmental influences underlying epigenetic changes. Key differences include the experimental methods used in animals, which allow for greater control over stress type and intensity, as well as the more frequent use of drugs, enabling the testing of hypotheses that can later be applied to human research.

However, knowledge about the functional impacts of DNA methylation variations is still in early stages. Smaller variations in DNA methylation are expected to have significant implications for the development of mental health disorders, particularly when compared with larger variations typically found in conditions such as cancer (Daskalakis and Yehuda 2014). Translational studies are essential to better understand this issue. The notion of epigenetic plasticity and the reversibility of epigenetically determined health conditions presents new opportunities for social and therapeutic interventions (Szyf 2012), including psychotherapy (Yehuda et al. 2013). Certain epigenetic changes associated with stress seem to be reversible, and a positive environment can induce variations that could have an impact on stress response (Morgan et al. 2013).

Dupras et al. (2019) discuss the relationship between epigenetics, ethics, law and society and find that, until 2012, studies were dominated by neutral and optimistic analyses; when, from then on, investigations turned to preventive analyses. By allowing a better understanding of the origins of the development of health and disease, epigenetics has been mobilized with optimism in the formulation of policies for the improvement of preventive strategies (Rial‐Sebbag et al. 2016). Social epigenetics has provided convincing arguments for the promotion of social policies when it demonstrates how first‐life experiences can influence gene expression later in life, inciting the development of policies to increase the future health of children (McBride and Koehly 2017; Park and Kobor 2015). These findings underscore the pivotal role of social determinants of health in preventing a range of health‐related conditions; for example, the potential epigenetic inheritance of obesity‐related diseases is now seen as a public health concern considering recent advances in epigenetics (Niculescu 2011). Moreover, they broaden the horizons for potential new directions in neuroscience (Lang et al. 2016).

Although aiming to be comprehensive, this scoping review must be considered with the following limitations, given the still early stage of knowledge arising from the intersection of epigenetics, protective/positive factors and physical/mental health. The primary limitation is that the decision to conduct a more comprehensive review is based on the inserted epigenetic studies, encompassing the heterogeneity in methodologies, sample populations (human and animal), epigenetic analysis techniques and wide diversity of physical and mental health problems, as well as various protective factors and factors that provide positive outcomes. This poses challenges in drawing definitive conclusions, introducing a real difficulty in categorizing such diversity with the necessary conciseness in the limited editorial size available. Such richness makes it impossible to describe each item in depth, limiting itself to pointing out possible relevance and paths for future studies. Another aspect is the absence of a standardized conceptualization of resilience, as it remains an ambiguous and inconsistent concept, operationalized differently across studies (Black et al. 2024). A similar challenge arises in differentiating between health‐promoting and protective factors. It remains unclear to what extent some positive exposures universally enhance health, while others may primarily function to counteract vulnerability. Future studies with a precise conceptual cut may be conducted by a meta‐analysis design, which may add the presented results. Additionally, there appears to be a notable risk of confounding variables in studies of environmental and social epigenetics. Furthermore, significant metaethical questions arise concerning the extent of normative and prescriptive value that can be attributed to empirical findings (Dupras et al. 2019; Chung et al. 2016; Huang and King 2017; Juengst et al. 2014). It is suggested that further studies monitor the physical/mental health of groups or populations, including epigenetic data, which could show a greater understanding of biological and environmental influences. On the other hand, the comprehensive nature of this scoping review, encompassing both empirical and theoretical works, is a significant strength, providing a broad overview of the current state of research and helping to identify gaps for future investigations.

## 5 Conclusion

This scoping review underscores the emerging significance of epigenetic mechanisms, particularly DNA methylation, in understanding resilience and protective/positive factors. The findings highlight the intricate interplay between epigenetic and environmental influences, shaping mental and physical health outcomes across the lifespan. It emphasizes the need for continued research, particularly in diverse populations, to unravel the complexities of gene‐environmental interactions and their implications for public health. The potential applications of these findings extend beyond scientific inquiry, offering insights into public health interventions and social policies. By identifying key protective/positive factors and their biological correlations, this research contributes to the development of evidence‐based strategies aimed at fostering resilience, reducing health inequalities and promoting well‐being and health across different population groups. As the field of social epigenetics continues to evolve, it holds promise for driving scientific, social and political advancements that can positively impact public health outcomes globally.

## Author Contributions

Simone Gonçalves de Assis participated in all stages; Joviana Quintes Avanci and Fernanda Serpeloni selected articles and drafted the text. Pedro Tavares participated in the selection stage and drafted the final product. Nayara Oliveira performed the document selection and epigenetic analyses.

## Conflicts of Interest

The authors declare no conflicts of interest.

## Data Availability

The data that support the findings of this study are available on request from the corresponding author. The data are not publicly available due to privacy or ethical restrictions.

## References

[jdn70042-bib-0001] Alisch, R. S. , C. Pankaj , A. S. Fox , et al. 2014. “Differentially Methylated Plasticity Genes in the Amygdala of Young Primates Are Linked to Anxious Temperament, an at Risk Phenotype for Anxiety and Depressive Disorders.” Journal of Neuroscience 34, no. 47: 15548–15556. 10.1523/JNEUROSCI.3338-14.2014. 25411484 PMC4236392

[jdn70042-bib-0002] Aristizabal, M. J. , I. Anreiter , T. Halldorsdottir , et al. 2020. “Biological Embedding of Experience: A Primer on Epigenetics.” Proceedings of the National Academy of Sciences of the United States of America 117, no. 38: 23261–23269. 10.1073/pnas.1820838116. 31624126 PMC7519272

[jdn70042-bib-0003] Assis, S. G. , R. P. Pesce , and J. Q. Avanci . 2006. “Resilience Emphasizing the Protection of Adolescents.” In Resilience Emphasizing the Protection of Adolescents, 144. Artmed.

[jdn70042-bib-0004] Babenko, O. , I. Kovalchuk , and G. A. S. Metz . 2015. “Stress‐Induced Perinatal and Transgenerational Epigenetic Programming of Brain Development and Mental Health.” Neuroscience and Biobehavioral Reviews 48: 70–91. 10.1016/j.neubiorev.2014.11.013. 25464029

[jdn70042-bib-0005] Barter, J. D. , and T. C. Foster . 2018. “Aging in the Brain: New Roles of Epigenetics in Cognitive Decline.” Neuroscientist 24, no. 5: 516–525. 10.1177/1073858418780971. 29877135

[jdn70042-bib-0006] Beach, S. R. , M. K. Lei , G. H. Brody , and R. A. Philibert . 2018. “Prevention of Early Substance Use Mediates, and Variation at SLC6A4 Moderates, SAAF Intervention Effects on OXTR Methylation.” Prevention Science 19, no. 1: 90–100. 10.1007/s11121-016-0709-5. 27655391 PMC5360555

[jdn70042-bib-0007] Binder, E. B. 2017. “Dissecting the Molecular Mechanisms of Gene x Environment Interactions: Implications for Diagnosis and Treatment of Stress‐Related Psychiatric Disorders.” European Journal of Psychotraumatology 8, no. sup5: 1412745. 10.1080/20008198.2017.1412745. 29372006 PMC5774411

[jdn70042-bib-0008] Binder, E. B. , and F. Holsboer . 2012. “Low Cortisol and Risk and Resilience to Stress‐Related Psychiatric Disorders.” Biological Psychiatry 71, no. 4: 282–283. 10.1016/j.biopsych.2011.12.008. 22265026

[jdn70042-bib-0009] Black, M. H. , J. Helander , J. Segers , et al. 2024. “Resilience in the Face of Neurodivergence: A Scoping Review of Resilience and Factors Promoting Positive Outcomes.” Clinical Psychology Review 113: 102487. 10.1016/j.cpr.2024.102487. 39178757

[jdn70042-bib-0010] Blaze, J. 2018. “Polyphenolic Compounds Alter Stress‐Induced Patterns of Global DNA Methylation in Brain and Blood.” Molecular Nutrition & Food Research 62, no. 8: e1700722. 10.1002/mnfr.201700722. 29473292 PMC5953514

[jdn70042-bib-0011] Boivin, M. J. , A. M. Kakooza , B. C. Warf , L. L. Davidson , and E. L. Grigorenko . 2015. “Reducing Neurodevelopmental Disorders and Disability Through Research and Interventions.” Nature 527, no. 7578: S155–S160. 10.1038/nature16029. 26580321

[jdn70042-bib-0012] Booij, L. , M. Szyf , A. Carballedo , et al. 2015. “DNA Methylation of the Serotonin Transporter Gene in Peripheral Cells and Stress‐Related Changes in Hippocampal Volume: A Study in Depressed Patients and Healthy Controls.” PLoS ONE 10, no. 3: e0119061. 10.1371/journal.pone.0119061. 25781010 PMC4363605

[jdn70042-bib-0013] Bove, R. M. , E. Patrick , C. M. Aubin , et al. 2018. “Reproductive Period and Epigenetic Modifications of the Oxidative Phosphorylation Pathway in the Human Prefrontal Cortex.” PLoS ONE 13, no. 7: e0199073. 10.1371/journal.pone.0199073. 30052629 PMC6063396

[jdn70042-bib-0014] Burns, S. B. , J. K. Szyszkowicz , G. N. Luheshi , P. E. Lutz , and G. Turecki . 2017. “Plasticity of the Epigenome During Early‐Life Stress.” In Seminars in Cell & Developmental Biology, vol. 77, 115–132. Academic Press. 10.1016/j.semcdb.2017.09.033. 29017800

[jdn70042-bib-0015] Burris, H. H. , A. A. Baccarelli , R. O. Wright , and R. J. Wright . 2016. “Epigenetics: Linking Social and Environmental Exposures to Preterm Birth.” Pediatric Research 79, no. 1–2: 136–140. 10.1038/pr.2015.191. 26460521 PMC4740247

[jdn70042-bib-0016] Cadet, J. L. 2016. “Epigenetics of Stress, Addiction, and Resilience: Therapeutic Implications.” Molecular Neurobiology 53, no. 1: 545–560. 10.1007/s12035-014-9040-y. 25502297 PMC4703633

[jdn70042-bib-0017] Canli, T. 2019 Dec. “A Model of Human Endogenous Retrovirus (HERV) Activation in Mental Health and Illness.” Medical Hypotheses 133: 109404. 10.1016/j.mehy.2019.109404. 31557593

[jdn70042-bib-0018] Cao‐Lei, L. , R. Massart , M. J. Suderman , et al. 2014. “DNA Methylation Signatures Triggered by Prenatal Maternal Stress Exposure to a Natural Disaster: Project Ice Storm.” PLoS ONE 9, no. 9: e107653. 10.1371/journal.pone.0107653. 25238154 PMC4169571

[jdn70042-bib-0019] Champagne, F. A. 2010. “Epigenetic Influence of Social Experiences Across the Lifespan.” Developmental Psychobiology 52, no. 4: 299–311. 10.1002/dev.20436. 20175106

[jdn70042-bib-0020] Champagne, F. A. 2013. “Early Environments, Glucocorticoid Receptors, and Behavioral Epigenetics.” Behavioral Neuroscience 127, no. 5: 628–636. 10.1037/a0034186. 24128352

[jdn70042-bib-0021] Champagne, F. A. , and J. P. Curley . 2008. “Maternal Regulation of Estrogen Receptor α Methylation.” Current Opinion in Pharmacology 8, no. 6: 735–739. 10.1016/j.coph.2008.06.018. 18644464 PMC2612119

[jdn70042-bib-0022] Champagne, F. A. , and J. P. Curley . 2011. “Epigenetic Influence of the Social Environment.” In Brain, Behavior and Epigenetics. Epigenetics and Human Health edited by A. Petronis and J. Mill , 185–208. Springer. 10.1007/978-3-642-17426-1.

[jdn70042-bib-0023] Chang, H. L. , M. Wang , W. Xia , et al. 2016. “Perinatal Exposure to Low‐Dose Bisphenol a Disrupts Learning/Memory and DNA Methylation of Estrogen Receptor Alpha in the hippocampus.” Toxicology Research 5, no. 3: 828–835. 10.1039/c5tx00449g. 30090393 PMC6060734

[jdn70042-bib-0024] Chen, X. , W. Liu , L. Wang , et al. 2012. “Psychosocial Functioning and Cognitive Deficits Are Not Associated With Membrane‐Bound Catechol‐O‐Methyltransferase Deoxyribonucleic Acid Methylation in Siblings of Patients With Schizophrenia.” Journal of Nervous and Mental Disease 200, no. 11: 941–945. 10.1097/NMD.0b013e3182718c35. 23124177

[jdn70042-bib-0025] Chen, X. , S. Nishitani , E. Haroon , A. K. Smith , and J. K. Rilling . 2019. “OXTR Methylation Modulates Exogenous Oxytocin Effects on Human Brain Activity During Social Interaction.” Genes, Brain, and Behavior 19, no. 1: e12555. 10.1111/gbb.12555. 30624029

[jdn70042-bib-0026] Chen, Y. , X. Li , I. Kobayashi , D. Tsao , and T. A. Mellman . 2016. “Expression and Methylation in Posttraumatic Stress Disorder and Resilience; Evidence of a Role for Odorant Receptors.” Psychiatry Research 245: 36–44. 10.1016/j.psychres.2016.07.045. 27526315 PMC5148136

[jdn70042-bib-0027] Chocyk, A. , I. Majcher‐Maślanka , D. Dudys , A. Przyborowska , and K. Wędzony . 2013. “Impact of Early‐Life Stress on the Medial Prefrontal Cortex Functions. A Search for the Pathomechanisms of Anxiety and Mood Disorders.” Pharmacological Reports 65, no. 6: 1462–1470. 10.1016/S1734-1140(13)71506-8. 24552993

[jdn70042-bib-0028] Chung, E. , J. Cromby , D. Papadopoulos , and C. Tufarelli . 2016. “Social Epigenetics: A Science of Social Science?” Sociological Review 64, no. Suppl. 1: 168–185. 10.1111/2059-7932.120.

[jdn70042-bib-0029] Ciernia, A. V. , B. I. Laufer , K. W. Dunaway , et al. 2018. “Experience‐Dependent Neuroplasticity of the Developing Hypothalamus: Integrative Epigenomic Approaches.” Epigenetics 13, no. 3: 318–330. 10.1080/15592294.2018.1451720. 29613827 PMC5997166

[jdn70042-bib-0030] Cirulli, F. 2017. “Interactions Between Early Life Stress and Metabolic Stress in Programming of Mental and Metabolic Health.” Current Opinion in Behavioral Sciences 14: 65–71. 10.1016/j.cobeha.2016.12.009.

[jdn70042-bib-0031] Connolly, S. L. , T. B. Stoop , M. W. Logue , et al. 2018. “Posttraumatic Stress Disorder Symptoms, Temperament, and the Pathway to Cellular Senescence.” Journal of Traumatic Stress 31, no. 5: 676–686. 10.1002/jts.22325. 30338579 PMC6197884

[jdn70042-bib-0032] Conradt, E. , K. Hawes , D. Guerin , et al. 2016. “The Contributions of Maternal Sensitivity and Maternal Depressive Symptoms to Epigenetic Processes and Neuroendocrine Functioning.” Child Development 87, no. 1: 73–85. 10.1111/cdev.12483. 26822444 PMC4733872

[jdn70042-bib-0033] Coopersmith, S. 1967. The Antecedents of Self‐Esteem. Freeman.

[jdn70042-bib-0034] Coutinho, J. , E. Ribeiro , R. Ferreirinha , and P. Dias . 2010. “Versão Portuguesa da Escala de Dificuldades de Regulação Emocional e Sua Relação com Sintomas Psicopatológicos.” Revista de Psiquiatria Clínica 37, no. 4: 145–151. 10.1590/S0101-60832010000400001.

[jdn70042-bib-0035] Cramer, T. , T. Rosenberg , T. Kisliouk , and N. Meiri . 2018. “Early‐Life Epigenetic Changes Along the Corticotropin‐Releasing Hormone (CRH) Gene Influence Resilience or Vulnerability to Heat Stress Later in Life.” Molecular Psychiatry 24, no. 7: 1013–1026. 10.1038/s41380-018-0280-5. 30742007

[jdn70042-bib-0036] Daskalakis, N. P. , and R. Yehuda . 2014. “Site‐Specific Methylation Changes in the Glucocorticoid Receptor Exon 1F Promoter in Relation to Life Adversity: Systematic Review of Contributing Factors.” Frontiers in Neuroscience 8: 369. 10.3389/fnins.2014.00369. 25484853 PMC4240065

[jdn70042-bib-0037] Del Campo, C. M. Z. , M. Martínez‐Rosas , and V. Guarner‐Lans . 2018. “Epigenetic Programming of Synthesis, Release, and/or Receptor Expression of Common Mediators Participating in the Risk/Resilience for Comorbid Stress‐Related Disorders and Coronary Artery Disease.” International Journal of Molecular Sciences 19, no. 4: 1224. 10.3390/ijms19041224. 29670001 PMC5979500

[jdn70042-bib-0038] Deng, J. H. , W. Yan , Y. Han , et al. 2017. “Predictable Chronic Mild Stress During Adolescence Promotes Fear Memory Extinction in Adulthood.” Scientific Reports 7, no. 1: 7857. 10.1038/s41598-017-08017-7. 28798340 PMC5552791

[jdn70042-bib-0039] Denk, F. , S. B. McMahon , and I. Tracey . 2014. “Pain Vulnerability: A Neurobiological Perspective.” Nature Neuroscience 17, no. 2: 192–200. 10.1038/nn.3628. 24473267

[jdn70042-bib-0040] Di Sante, J. , E. Ismaylova , Z. Nemoda , J. P. Gouin , W. J. Yu , and W. Caldwell . 2018. “Peripheral DNA Methylation of HPA Axis‐Related Genes in Humans: Cross‐Tissue Convergence, Two‐Year Stability, and Behavioural and Neural Correlates.” Psychoneuroendocrinology 97: 196–205. 10.1016/j.psyneuen.2018.07.019. 30059826

[jdn70042-bib-0041] Díaz‐Anzaldúa, A. , A. Díaz‐Martínez , and L. R. Díaz‐Martínez . 2011. “The Complex Interplay of Genetics, Epigenetics, and Environment in the Predisposition to Alcohol Dependence.” Salud Ment [Revista en la Internet] 34, no. 2: 157–166. http://www.scielo.org.mx/scielo.php?script=sci_arttext&pid=S0185‐33252011000200009&lng=es.

[jdn70042-bib-0042] Duclot, F. , and M. Kabbaj . 2013. “Individual Differences in Novelty Seeking Predict Subsequent Vulnerability to Social Defeat Through a Differential Epigenetic Regulation of Brain‐Derived Neurotrophic Factor Expression.” Journal of Neuroscience 33, no. 27: 11048–11060. 10.1523/JNEUROSCI.0199-13.2013. 23825410 PMC3718363

[jdn70042-bib-0043] Duclot, F. , and M. Kabbaj . 2015. “Epigenetic Mechanisms Underlying the Role of Brain‐Derived Neurotrophic Factor in Depression and Response to Antidepressants.” Journal of Experimental Biology 218, no. 1: 21–31. 10.1242/jeb.107086. 25568448 PMC4286703

[jdn70042-bib-0044] Dudley, K. J. , X. Li , M. S. Kobor , T. E. Kippin , and T. W. Bredy . 2011. “Epigenetic Mechanisms Mediating Vulnerability and Resilience to Psychiatric Disorders.” Neuroscience and Biobehavioral Reviews 35, no. 7: 1544–1551. 10.1016/j.neubiorev.2010.12.016. 21251925

[jdn70042-bib-0045] Duman, E. A. , and T. Canli . 2015. “Influence of Life Stress, 5‐HTTLPR Genotype, and SLC6A4 Methylation on Gene Expression and Stress Response in Healthy Caucasian Males.” Biol Mood Anxiety Disord 5: 2. 10.1186/s13587-015-0017-x. 25995833 PMC4438516

[jdn70042-bib-0046] Dupras, C. , K. M. Saulnier , and Y. Joly . 2019. “Epigenetics, Ethics, Law, and Society: A Multidisciplinary Review of Descriptive, Instrumental, Dialectical and Reflexive Analyses.” Social Studies of Science 49, no. 5: 785–810. 10.1177/0306312719866007. 31366289 PMC6801799

[jdn70042-bib-0047] Ebner, N. C. , T. Lin , M. Muradoglu , et al. 2019. “Associations Between Oxytocin Receptor Gene (OXTR) Methylation, Plasma Oxytocin, and Attachment Across Adulthood.” International Journal of Psychophysiology 136: 22–32. 10.1016/j.ijpsycho.2018.01.008. 29410310 PMC6072619

[jdn70042-bib-0048] Ehlert, U. 2013. “Enduring Psychobiological Effects of Childhood Adversity.” Psychoneuroendocrinology 38, no. 9: 1850–1857. 10.1016/j.psyneuen.2013.06.007. 23850228

[jdn70042-bib-0049] Elliott, E. , G. Ezra‐Nevo , L. Regev , A. Neufeld‐Cohen , and A. Chen . 2010. “Resilience to Social Stress Coincides With Functional DNA Methylation of the Crf Gene in Adult Mice.” Nature Neuroscience 13, no. 11: 1351–1353. 10.1038/nn.2642. 20890295

[jdn70042-bib-0050] Esposito, E. A. , M. J. Jones , J. R. Doom , J. L. MacIsaac , M. R. Gunnar , and M. S. Kobor . 2016. “Differential DNA Methylation in Peripheral Blood Mononuclear Cells in Adolescents Exposed to Significant Early but Not Later Childhood Adversity.” Development and Psychopathology 28, no. 4pt2: 1385–1399. 10.1017/S0954579416000055. 26847422 PMC5903568

[jdn70042-bib-0051] Folger, A. T. , L. Ding , H. Ji , et al. 2019. “Neonatal NR3C1 Methylation and Social‐Emotional Development at 6 and 18 Months of Age.” Frontiers in Behavioral Neuroscience 13: 14. 10.3389/fnbeh.2019.00014. 30804765 PMC6371639

[jdn70042-bib-0052] Folguera‐Blasco, N. 2018. “Epigenetic Regulation of Cell Fate Reprogramming in Aging and Disease: A Predictive Computational Model.” PLoS Computational Biology 14, no. 15: e1006052. 10.1371/journal.pcbi.1006052. 29543808 PMC5871006

[jdn70042-bib-0053] Franklin, T. B. , B. J. Saab , and I. M. Mansuy . 2012. “Neural Mechanisms of Stress Resilience and Vulnerability.” Neuron 75, no. 5: 747–761. 10.1016/j.neuron.2012.08.016. 22958817

[jdn70042-bib-0054] Fries, G. R. , M. P. Vasconcelos‐Moreno , C. Gubert , et al. 2014. “Hypothalamic‐Pituitary‐Adrenal Axis Dysfunction and Illness Progression in Bipolar Disorder.” International Journal of Neuropsychopharmacology 18, no. 1: pyu043. 10.1093/ijnp/pyu043. Erratum in: Int J Neuropsychopharmacol. 2016 Apr 27;19(10):pyw031. 25522387 PMC4368875

[jdn70042-bib-0055] Gemmel, M. , E. Bögi , C. Ragan , M. Hazlett , M. Dubovicky , and D. L. van den Hove . 2018. “Perinatal Selective Serotonin Reuptake Inhibitor Medication (SSRI) Effects on Social Behaviors, Neurodevelopment, and the Epigenome.” Neuroscience and Biobehavioral Reviews 85: 102–116. 10.1016/j.neubiorev.2017.04.023. 28472631

[jdn70042-bib-0056] Griffiths, B. B. , and R. G. Hunter . 2014. “Neuroepigenetics of Stress.” Neuroscience 275: 420–435. 10.1016/j.neuroscience.2014.06.041. 24976514

[jdn70042-bib-0057] Hammels, C. , J. Prickaerts , G. Kenis , T. Vanmierlo , M. Fischer , and H. W. Steinbusch . 2015. “Differential Susceptibility to Chronic Social Defeat Stress Relates to the Number of Dnmt3a‐Immunoreactive Neurons in the Hippocampal Dentate Gyrus.” Psychoneuro 51: 547–556. 10.1016/j.psyneuen.2014.09.021. 25445743

[jdn70042-bib-0058] Hertzman, C. 2012. “Putting the Concept of Biological Embedding in Historical Perspective.” Proceedings of the National Academy of Sciences of the United States of America 109, no. sup2: 17160–17167. 10.1073/pnas.1202203109. 23045673 PMC3477385

[jdn70042-bib-0059] Hertzman, C. 2013. “Inégalités Sociales de Santé, Développement de la Petite Enfance et Incorporation Biologique.” Revue d'Épidémiologie et de Santé Publique 61: S39–S46. 10.1016/j.respe.2013.03.036. 23684106

[jdn70042-bib-0060] Hiebel, N. , M. Rabe , K. Maus , F. Peusquens , L. Radbruch , and F. Geiser . 2021. “Resilience in Adult Health Science Revisited—A Narrative Review Synthesis of Process‐Oriented Approaches.” Frontiers in Psychology 12: 659395. 10.3389/fpsyg.2021.659395. 34149549 PMC8210849

[jdn70042-bib-0061] Hoffmann, F. , K. Allers , T. Rombey , et al. 2021. “Nearly 80 Systematic Reviews Were Published Each Day: Observational Study on Trends in Epidemiology and Reporting Over the Years 2000‐2019.” Journal of Clinical Epidemiology 138: 1–11. 10.1016/j.jclinepi.2021.05.022. 34091022

[jdn70042-bib-0062] Högfelt, T. , S. Barde , Z. Q. D. Xu , E. Kuteeva , J. Rüegg , and E. Le Maitre . 2018. “Neuropeptide, and Small Transmitter Coexistence: Fundamental Studies and Relevance to Mental Illness.” Frontiers in Neural Circuits 12: 106. 10.3389/fncir.2018.00106. 30627087 PMC6309708

[jdn70042-bib-0063] Holmes, L. , C. Chinaka , H. Elmi , et al. 2019. “Implication of Spiritual Network Support System in Epigenomic Modulation and Health Trajectory.” International Journal of Environmental Research and Public Health 16, no. 21: 4123. 10.3390/ijerph16214123. 31717711 PMC6862316

[jdn70042-bib-0064] Holt‐Lunstad, J. 2019. “Oxytocin, Social Relationships, and Health: An Introduction to the Special Issue.” International Journal of Psychophysiology 136: 1–4. 10.1016/j.ijpsycho.2018.12.008. 30653995

[jdn70042-bib-0065] Horvath, S. , and K. Raj . 2018. “DNA Methylation‐Based Biomarkers and the Epigenetic Clock Theory of Ageing.” Nature Reviews. Genetics 19, no. 6: 371–384. 10.1038/s41576-018-0004-3. 29643443

[jdn70042-bib-0066] Howie, H. 2019. “A Review of Epigenetic Contributions to Post‐Traumatic Stress Disorder.” Dialogues in Clinical Neuroscience 21, no. 4: 417–428. 10.31887/DCNS.2019.21.4/kressler. 31949409 PMC6952751

[jdn70042-bib-0067] Huang, J. Y. , and N. B. King . 2017. “Epigenetics Changes Nothing: What a New Scientific Field Does and Does Not Mean for Ethics and Social Justice.” Public Health Ethics 11, no. 1: 69–81. 10.1093/phe/phx013. 30619507 PMC6307350

[jdn70042-bib-0068] Ismaylova, E. , J. Di Sante , M. Szyf , et al. 2017. “Text Serotonin Transporter Gene Promoter Methylation in Peripheral Cells in Healthy Adults: Neural Correlates and Tissue Specificity.” European Neuropsychopharmacology 27, no. 10: 1032–1041. 10.1016/j.euroneuro.2017.07.005. 28774705

[jdn70042-bib-0069] Jakob, S. , K. Schraut , A. G. Schmitt , et al. 2014. “Differential Effects of Prenatal Stress in Female 5‐HTT‐Deficient Mice: Towards Molecular Mechanisms of Resilience.” Developmental Neuroscience 36: 454–464. 10.1159/000363695. 25195605

[jdn70042-bib-0070] Jawaid, A. , and I. M. Mansuy . 2019. “Inter‐ and Transgenerational Inheritance of Behavioral Phenotypes.” Current Opinion in Behavioral Sciences 25: 96–101. 10.1016/j.cobeha.2018.12.004.

[jdn70042-bib-0071] Jessen, H. M. , and A. P. Auger . 2011. “Sex Differences in Epigenetic Mechanisms may Underlie Risk and Resilience for Mental Health Disorders.” Epigenetics 6, no. 7: 857–861. 10.4161/epi.6.7.16517. 21617370 PMC3154426

[jdn70042-bib-0072] Jiang, S. , L. Postovit , A. Cattaneo , E. B. Binder , and K. J. Aitchison . 2019. “Epigenetic Modifications in Stress Response Genes Associated With Childhood Trauma.” Frontiers in Psychiatry 10: 808. 10.3389/fpsyt.2019.00808. 31780969 PMC6857662

[jdn70042-bib-0073] Jirtle, R. L. , and M. K. Skinner . 2007. “Environmental Epigenomics and Disease Susceptibility.” Nature Reviews. Genetics 8, no. 4: 253–262. 10.1038/nrg2045. PMC5940010 17363974

[jdn70042-bib-0074] Juengst, E. T. , J. R. Fishman , M. L. McGowan , and R. A. Settersten . 2014. “Serving Epigenetics Before Its Time.” Trends in Genetics 30, no. 10: 427–429. 10.1016/j.tig.2014.08.001. 25242336

[jdn70042-bib-0075] Kaufman, J. , J. L. Montalvo‐Ortiz , H. Holbrook , K. O'Loughlin , C. Orr , and C. Kearney . 2018. “Adverse Childhood Experiences, Epigenetic Measures, and Obesity in Youth.” Journal of Pediatrics 202: 150–156. 10.1016/j.jpeds.2018.06.051. 30177354 PMC6513669

[jdn70042-bib-0076] Kaufman, J. , N. F. Wymbs , J. L. Montalvo‐Ortiz , et al. 2018. “Methylation in OTX2 and Related Genes, Maltreatment, and Depression in Children.” Neuropsychopharmacol 43, no. 11: 2204–2211. 10.1038/s41386-018-0157-y. PMC6135753 30089883

[jdn70042-bib-0077] Kaye‐Kauderer, H. , J. Feingold , A. Feder , S. Southwick , and D. Charney . 2021. “Resilience in the Age of COVID‐19.” BJPsych Advances 27: 166–178. 10.1192/bja.2021.5.

[jdn70042-bib-0078] Kim, T. Y. , S. J. Kim , H. G. Chung , J. H. Choi , S. H. Kim , and J. I. Kang . 2017. “Epigenetic Alterations of the BDNF Gene in Combat‐Related Post‐Traumatic Stress Disorder.” Acta Psychiatrica Scandinavica 135, no. 2: 170–179. 10.1111/acps.12675. 27886370

[jdn70042-bib-0079] Kingston, D. , M. K. Mughal , M. Arshad , I. Kovalchuk , G. A. Metz , and K. Wynne‐Edwards . 2019. “Prediction and Understanding of Resilience in Albertan Families: Longitudinal Study of Disaster Responses (PURLS)–Protocol.” Frontiers in Psychiatry 10: 729. 10.3389/fpsyt.2019.00729. 31736793 PMC6834684

[jdn70042-bib-0080] Kisliouk, T. , T. Cramer , and N. Meiri . 2017. “Methyl Cpg Level at Distal Part of Heat‐Shock Protein Promoter HSP 70 Exhibits Epigenetic Memory for Heatstress by Modulating Recruitment of POU 2F1‐Associated Nucleosome‐Remodeling Deacetylase (Nu RD) Complex.” Journal of Neurochemistry 141, no. 3: 358–372. 10.1111/jnc.14014. 28278364

[jdn70042-bib-0081] Klengel, T. , J. Pape , E. B. Binder , and D. Mehta . 2014. “The Role of DNA Methylation in Stress‐Related Psychiatric Disorders.” Neuropharmacology 80: 115–132. 10.1016/j.neuropharm.2014.01.013. 24452011

[jdn70042-bib-0082] Kogan, S. M. , J. Cho , S. R. Beach , A. K. Smith , and S. Nishitani . 2018. “Oxytocin Receptor Gene Methylation and Substance Use Problems Among Young African American Men.” Drug and Alcohol Dependence 192: 309–315. 10.1016/j.drugalcdep.2018.08.022. 30308385 PMC6202060

[jdn70042-bib-0083] Krol, K. M. , R. G. Moulder , T. S. Lillard , T. Grossmann , and J. J. Connelly . 2019. “Epigenetic Dynamics in Infancy and the Impact of Maternal Engagement.” Science Advances 5, no. 10: eaay0680. 10.1126/sciadv.aay0680. 31663028 PMC6795517

[jdn70042-bib-0084] Krol, K. M. , M. H. Puglia , J. P. Morris , J. J. Connelly , and T. Grossmann . 2019. “Epigenetic Modification of the Oxytocin Receptor Gene Is Associated With Emotion Processing in the Infant Brain.” Developmental Cognitive Neuroscience 37: 100648. 10.1016/j.dcn.2019.100648. 31125951 PMC6969294

[jdn70042-bib-0085] Labonté, B. , Y. H. Jeong , E. Parise , et al. 2019. “Gadd45b Mediates Depressive‐Like Role Through DNA Demethylation.” Scientific Reports 9, no. 1: 1–9. 10.1038/s41598-019-40844-8. 30874581 PMC6420662

[jdn70042-bib-0086] Lang, T. , M. Kelly‐Irving , S. Lamy , B. Lepage , and C. Delpierr . 2016. “Construction de la Santé et des Inégalités Sociales de Santé: Les Gènes contre les Déterminants Sociaux? [Construction of Health and Social Inequalities of Health: Genes Against Social Determinants?].” Santé Publique 28, no. 2: 169–179. 27392051

[jdn70042-bib-0087] Lapp, H. E. , S. Ahmed , C. L. Moore , and R. G. Hunter . 2019. “Toxic Stress History and Hypothalamic‐Pituitary‐Adrenal axis Function in a Social Stress Task: Genetic and Epigenetic Factors.” Neurotoxicology and Teratology 71: 41–49. 10.1016/j.ntt.2018.01.011. 29475055

[jdn70042-bib-0088] Lee, A. G. , C. L. Buckmaster , E. Yi , A. F. Schatzberg , and D. M. Lyons . 2014. “Coping and Glucocorticoid Receptor Regulation by Stress Inoculation.” Psychoneuroendocrinology 49: 272–279. 10.1016/j.psyneuen.2014.07.020. 25127085 PMC4165807

[jdn70042-bib-0089] Lucero, I. 2018. “Written in the Body?” Journal of Child and Adolescent Trauma 11, no. 4: 443–455. 10.1007/s40653-018-0205-0. 32318167 PMC7163842

[jdn70042-bib-0090] Lupien, S. J. , B. S. Mcewen , M. R. Gunnar , and C. Heim . 2009. “Effects of Stress Throughout the Lifespan on the Brain.” Nature Reviews. Neuroscience 10, no. 6: 434–445. 10.1038/nrn2639. 19401723

[jdn70042-bib-0091] Maccari, S. , H. J. Krugers , S. Morley‐Fletcher , M. Szyf , and P. J. Brunton . 2014. “The Consequences of Early‐Life Adversity: Neurobiological, Behavioural, and Epigenetic Adaptations.” Journal of Neuroendocrinology 26, no. 10: 707–723. 10.1111/jne.12175. 25039443

[jdn70042-bib-0092] Martin, C. L. , L. Ghastine , E. K. Lodge , R. Dhingra , and C. K. Ward‐Caviness . 2022. “Understanding Health Inequalities Through the Lens of Social Epigenetics.” Annual Review of Public Health 43: 235–254. 10.1146/annurev-publhealth-052020-105613. PMC9584166 35380065

[jdn70042-bib-0093] Mattern, F. , A. Post , F. Solger , et al. 2019. “Prenatal and Postnatal Experiences Associated With Epigenetic Changes in the Adult Mouse Brain.” Behavioural Brain Research 359: 143–148. 10.1016/j.bbr.2018.10.037. 30385366

[jdn70042-bib-0094] Maud, C. , J. Ryan , J. E. McIntosh , and C. A. Olsson . 2018. “The Role of Oxytocin Receptor Gene (OXTR) DNA Methylation (mDNA) in Human Social and Emotional Functioning: A Systematic Narrative Review.” BMC Psychiatry 18, no. 1: 154. 10.1186/s12888-018-1740-9. 29843655 PMC5975530

[jdn70042-bib-0095] McBride, C. M. , and L. M. Koehly . 2017. “Imagining Roles for Epigenetics in Health Promotion Research.” Journal of Behavioral Medicine 40, no. 2: 229–238. 10.1007/s10865-016-9764-4. 27412775 PMC5332486

[jdn70042-bib-0096] McGuinness, D. , S. Mohammed , L. Monaghan , et al. 2018. “A Molecular Signature for Delayed Graft Function.” Aging Cell 17, no. 5: e12825. 10.1111/acel.12825. 30094915 PMC6156499

[jdn70042-bib-0097] Mehta, D. , D. Bruenig , T. Carrillo‐Roa , et al. 2017. “Genomewide DNA Methylation Analysis in Combat Veterans Reveals a Novel Locus for PTSD.” Acta Psychiatrica Scandinavica 136, no. 5: 493–505. 10.1111/acps.12778. 28795405

[jdn70042-bib-0098] Mehta, D. , D. Bruenig , B. Lawford , W. Harvey , T. Carrillo‐Roa , and C. P. Morris . 2018. “Accelerated DNA Methylation Aging and Increased Resilience in Veterans: The Biological Cost for Soldiering On.” Neurobiology of Stress 8: 112–119. 10.1016/j.ynstr.2018.04.001. 29888306 PMC5991315

[jdn70042-bib-0099] Mehta, D. , O. Miller , D. Bruenig , G. David , and J. Shakespeare‐Finch . 2020. “A Systematic Review of DNA Methylation and Gene Expression Studies in Posttraumatic Stress Disorder, Posttraumatic Growth, and Resilience.” Journal of Traumatic Stress 33, no. 2: 171–180. 10.1002/jts.22472. 31951051

[jdn70042-bib-0100] Milaniak, I. , C. A. Cecil , E. D. Barker , et al. 2017. “Variation in Methylation of the Oxytocin Receptor Gene Predicts Children's Resilience to Prenatal Stress.” Development and Psychopathology 29, no. 5: 1663–1674. 10.1017/S0954579417001316. 29162179

[jdn70042-bib-0101] Miller, G. E. , T. Yu , E. Chen , and G. H. Brody . 2015. “Self‐Control Forecasts Better Psychosocial Outcomes but Faster Epigenetic Aging in low‐SES Youth.” Proceedings. National Academy of Sciences. United States of America 112, no. 33: 10325–10330. 10.1073/pnas.1505063112. PMC4547243 26170291

[jdn70042-bib-0102] Morgan, C. , M. O'Donovan , R. A. Bittner , et al. 2013. “How Can Risk and Resilience Factors Be Leveraged to Optimize Discovery Pathways?” In Schizophrenia: Evolution and Synthesis. Cambridge: MIT Press. 33886220

[jdn70042-bib-0103] Murgatroyd, C. , and D. Spengler . 2012. “Genetic Variation in the Epigenetic Machinery and Mental Health.” Current Psychiatry Reports 14, no. 2: 138–149. 10.1007/s11920-012-0255-1. 22307409

[jdn70042-bib-0104] Nabeshima, T. , and H. Kim . 2013. “Involvement of Genetic and Environmental Factors in the Onset of Depression.” Experimental Neurobiology 22, no. 4: 235–243. 10.5607/en.2013.22.4.235. 24465138 PMC3897684

[jdn70042-bib-0105] Niculescu, M. 2011. “Epigenetic Transgenerational Inheritance: Should Obesity‐Prevention Policies Be Reconsidered?” Synesis: A Journal of Science, Technology, Ethics, and Policy 2, no. 1: G18–G26.

[jdn70042-bib-0106] Noro, G. , and M. C. C. Gon . 2015. “Epigenetics, Maternal Care, and Vulnerability to Stress: Basic Concepts and Applicability.” Psychology 28, no. 4: 829–839. 10.1590/1678-7153.201528422.

[jdn70042-bib-0107] Ohm, J. E. 2019. “Environmental Exposures, the Epigenome, and African American Women's Health.” Journal of Urban Health 96, no. 1: 50–56. 10.1007/s11524-018-00332-2. 30488361 PMC6430284

[jdn70042-bib-0108] Pan‐Vazquez, A. , N. Rye , M. Ameri , et al. 2015. “Impact of Voluntary Exercise and Housing Conditions on Hippocampal Glucocorticoid Receptor, miR‐124 and Anxiety.” Molecular Brain 8: 40. 10.1186/s13041-015-0128-8. 26135882 PMC4487841

[jdn70042-bib-0109] Papadopoulou, Z. , A. M. Vlaikou , D. Theodoridou , et al. 2019. “Stressful Newborn Memories: Pre‐Conceptual, in Utero, and Postnatal Events.” Frontiers in Psychiatry 10: 220. 10.3389/fpsyt.2019.00220. 31057437 PMC6482218

[jdn70042-bib-0110] Park, M. , and M. S. Kobor . 2015. “The Potential of Social Epigenetics for Child Health Policy.” Canadian Public Policy/Analyse de Politiques 41, no. Suppl. 2: S89–S96. 10.3138/cpp.2014-081.

[jdn70042-bib-0111] Pfeiffer, J. R. , L. Mutesa , and M. Uddin . 2018. “Traumatic Stress Epigenetics.” Current Behavioral Neuroscience Reports 5, no. 1: 81–93. 10.1007/s40473-018-0143-z. 30225184 PMC6138446

[jdn70042-bib-0112] Plomin, R. , and M. A. Simpson . 2013. “The Future of Genomics for Developmentalists.” Development and Psychopathology 25, no. 4 Pt 2: 1263–1278. 10.1017/S0954579413000606. 24342839 PMC3967388

[jdn70042-bib-0113] Pluess, M. 2017. “Vantage Sensitivity: Environmental Sensitivity to Positive Experiences as a Function of Genetic Differences.” Journal of Personality 85: 38–50. 10.1111/jopy.12218. 26271007

[jdn70042-bib-0114] Puglia, M. H. , J. J. Connelly , and J. P. Morris . 2018. “Epigenetic Regulation of the Receptor Oxytocin Is Associated With Neural Response During Selective Social Attention.” Translational Psychiatry 8, no. 1: 1–10. 10.1038/s41398-018-0159-x. 29907738 PMC6003910

[jdn70042-bib-0115] Puglia, M. H. , T. S. Lillard , J. P. Morris , and J. J. Connelly . 2015. “Epigenetic Modification of the Oxytocin Receptor Gene Influences the Perception of Anger and Fear in the Human Brain.” Proceedings. National Academy of Sciences. United States of America 112, no. 11: 3308–3313. 10.1073/pnas.1422096112. PMC4372000 25675509

[jdn70042-bib-0116] Rial‐Sebbag, E. , L. C. Guibet , and U. Simeoni . 2016. “DOHaD and Epigenetic Information: Societal Challenges.” Medicine and Science 32, no. 1: 100–105. 10.1051/medsci/20163201016. 26850614

[jdn70042-bib-0117] Roth, T. L. 2013. “Epigenetic Mechanisms in the Development of Behavior: Advances, Challenges, and Future Promises of a New Field.” Development and Psychopathology 25, no. 4pt2: 1279–1291. 10.1017/S0954579413000618. 24342840 PMC4080409

[jdn70042-bib-0118] Roth, T. L. , and J. David Sweatt . 2011. “Annual Research Review: Epigenetic Mechanisms and Environmental Shaping of the Brain During Sensitive Periods of Development.” Journal of Child Psychology and Psychiatry 52, no. 4: 398–408. 10.1111/j.1469-7610.2010.02282.x. 20626526 PMC2965301

[jdn70042-bib-0119] Rusiecki, J. A. , L. Chen , V. Srikantan , et al. 2012. “DNA Methylation in Repetitive Elements and Post‐Traumatic Stress Disorder: A Case–Control Study of US Military Service Members.” Epigenomics 4, no. 1: 29–40. 10.2217/epi.11.116. 22332656 PMC3809831

[jdn70042-bib-0120] Rutten, B. P. F. , C. Hammels , N. Geschwind , et al. 2013 Jul. “Resilience in Mental Health: Linking Psychological and Neurobiological Perspectives.” Acta Psychiatrica Scandinavica 128, no. 1: 3–20. 10.1111/acps.12095. 23488807 PMC3746114

[jdn70042-bib-0121] Rutten, B. P. F. , E. Vermetten , C. H. Vinkers , et al. 2018. “Longitudinal Analyses of the DNA Methylome in Deployed Military Servicemen Identify Susceptibility Loci for Post‐Traumatic Stress Disorder.” Molecular Psychiatry 23: 1145–1156. 10.1038/mp.2017.120. 28630453 PMC5984086

[jdn70042-bib-0122] Schiele, M. A. , and K. Domschke . 2018. “Epigenetics at the Crossroads Between Genes, Environment, and Resilience in Anxiety Disorders.” Genes, Brain, and Behavior 17, no. 3: e12423. 10.1111/gbb.12423. 28873274

[jdn70042-bib-0123] Schiele, M. A. , M. G. Gottschalk , and K. Domschke . 2020. “The Applied Implications of Epigenetics in Anxiety, Affective and Stress‐Related Disorders—A Review and Synthesis on Psychosocial Stress, Psychotherapy and Prevention.” Clinical Psychology Review 77: 101830. 10.1016/j.cpr.2020.101830. 32163803

[jdn70042-bib-0125] Serpeloni, F. , K. M. Radtke , T. Hecker , et al. 2016. “Epigenetic Biomarkers of Prenatal Maternal Stress.” Epigenetics and Neuroendocrinology: Clinical Focus on Psychiatry 2: 177–196. 10.1007/978-3-319-29901-3_8.

[jdn70042-bib-0124] Serpeloni, F. , K. M. Radtke , T. Hecker , et al. 2019. “Does Prenatal Stress Shape Postnatal Resilience? An Epigenome‐Wide Study on Violence and Mental Health in Humans.” Frontiers in Genetics 10: 269. 10.3389/fgene.2019.00269. 31040859 PMC6477038

[jdn70042-bib-0126] Sevane, N. , R. Martínez , and M. W. Bruford . 2018. “Genome‐Wide Differential DNA Methylation in Tropically Adapted Creole Cattle and Their Iberian Ancestors.” Animal Genetics 50, no. 1: 15–26. 10.1111/age.12731. 30565712

[jdn70042-bib-0127] Sharma, S. 2019. “Genomic Updates in Understanding PTSD.” Progress in Neuro‐Psychopharmacology & Biological Psychiatry 90: 197–203. 10.1016/j.pnpbp.2018.11.010. 30452941 PMC6431237

[jdn70042-bib-0128] Sherbourne, C. D. , and A. L. Stewart . 1991. “The MOS Social Support Survey.” Social Science & Medicine 32: 705–714. 10.1016/0277-9536(91)90150-B. 2035047

[jdn70042-bib-0129] Shields, A. E. 2017. “Epigenetic Signals of How Social Disadvantage “Gets Under the Skin”: A Challenge to the Public Health Community.” Epigenomics 9, no. 3: 223–229. 10.2217/epi-2017-0013. 28234017

[jdn70042-bib-0130] Silva, A. S. 2018. “Maternal Exposure to Fluoxetine During Gestation and Lactation Induces Long‐lasting Changes in the DNA Methylation Profile of Offspring's Brain and Affects the Social Interaction of Rat.” Brain Research Bulletin 142: 409–413. 10.1016/j.brainresbull.2018.09.007. 30236534

[jdn70042-bib-0131] Sipahi, L. , M. Uddin , Z. C. Hou , et al. 2014. “Ancient Evolutionary Origins of Epigenetic Regulation Associated With Posttraumatic Stress Disorder.” Frontiers in Human Neuroscience 8: 284. 10.3389/fnhum.2014.00284. 24860472 PMC4026723

[jdn70042-bib-0132] Smeeth, D. , S. Beck , E. G. Karam , and M. Pluess . 2021. “The Role of Epigenetics in Psychological Resilience.” Lancet Psychiatry 8, no. 7: 620–629. 10.1016/S2215-0366(20)30515-0. 33915083 PMC9561637

[jdn70042-bib-0133] Stover, P. J. , W. P. T. James , A. Krook , and C. Garza . 2018. “Emerging Concepts on the Role of Epigenetics in the Relationships Between Nutrition and Health.” Journal of Internal Medicine 284, no. 1: 37–49. 10.1111/joim.12768. 29706028

[jdn70042-bib-0134] Surkan, P. J. , X. Hong , B. Zhang , et al. 2019. “Can Social Support During Pregnancy Affect Maternal DNA Methylation? Findings From a Cohort of African Americans.” Pediatric Research 88, no. 1: 131–138. 10.1038/s41390-019-0512-7. 31349361 PMC6982603

[jdn70042-bib-0135] Szyf, M. 2012. “The Early‐Life Social Environment and DNA Methylation.” Clinical Genetics 81, no. 4: 341–349. 10.1111/j.1399-0004.2012.01843.x. 22236068

[jdn70042-bib-0136] Taff, C. C. , L. Campagna , and M. N. Vitousek . 2019. “Genome‐Wide Variation in DNA Methylation Is Associated With Stress Resilience and Plumage Brightness in a Wild Bird.” Molecular Ecology 28, no. 16: 3722–3737. 10.1111/mec.15186. 31330076

[jdn70042-bib-0137] Teicher, M. H. , S. L. Andersen , A. Polcarib , C. M. Anderson , C. P. Navalta , and D. M. Kim . 2003. “The Neurobiological Consequences of Early Stress and Childhood Maltreatment.” Neuroscience and Biobehavioral Reviews 27: 33–44. 10.1016/S0149-7634(03)00007-1. 12732221

[jdn70042-bib-0138] Theilmann, W. , A. Kleimann , M. Rhein , et al. 1642. “Behavioral Differences of Male Wistar Rats From Different Vendors in Vulnerability and Resilience to Chronic Mild Stress Are Reflected in Epigenetic Regulation and Expression of p11.” Brain Research 2016: 505–515. 10.1016/j.brainres.2016.04.041. 27103570

[jdn70042-bib-0139] Tian, F. , and C. J. Marsit . 2018. “Environmentally Induced Epigenetic Plasticity in Development: Epigenetic Toxicity and Epigenetic Adaptation.” Current Epidemiology Reports 5, no. 4: 450–460. 10.1007/s40471-018-0175-7. 30984515 PMC6456900

[jdn70042-bib-0140] Tricco, A. C. , E. Lillie , W. Zarin , et al. 2018. “PRISMA Extension for Scoping Reviews (PRISMA‐ScR): Checklist and Explanation.” Annals of Internal Medicine 169, no. 7: 467–473. 10.7326/M18-0850. 30178033

[jdn70042-bib-0141] Tunc‐Ozcan, E. , L. J. Sittig , K. M. Harper , E. N. Graf , and E. E. Redei . 2014. “Hypothesis: Genetic and Epigenetic Risk Factors Interact to Modulate Vulnerability and Resilience to FASD.” Frontiers in Genetics 5: 261. 10.3389/fgene.2014.00261. 25140173 PMC4122175

[jdn70042-bib-0142] Tyagi, E. , Y. Zhuang , R. Agrawal , Z. Ying , and F. Gomez‐Pinilla . 2015. “Interactive Actions of *Bdnf* Methylation and Cell Metabolism for Building Neural Resilience Under the Influence of Diet.” Neurobiology of Disease 73: 307–318. 10.1016/j.nbd.2014.09.014. 25283985 PMC4754354

[jdn70042-bib-0143] Uddin, M. , S. Galea , S. C. Chang , et al. 2011. “Gene Expression and Methylation Signatures of MAN2C1 Are Associated With PTSD.” Disease Markers 30, no. 2–3: 111–121. 10.3233/DMA-2011-0750. 21508515 PMC3188659

[jdn70042-bib-0144] Uddin, M. , L. Sipahi , J. Li , and K. C. Koenen . 2013. “Sex Differences in DNA Methylation may Contribute to Risk of PTSD and Depression: A Review of Existing Evidence.” Depression and Anxiety 30, no. 12: 1151–1160. PMID: 24038555. 10.1002/da.22167. 23959810 PMC4530966

[jdn70042-bib-0145] Vidrascu, E. M. , A. C. Bashore , T. D. Howard , and J. B. Moore . 2019. “Effects of Early‐ and Mid‐Life Stress on DNA Methylation of Genes Associated With Subclinical Cardiovascular Disease and Cognitive Impairment: A Systematic Review.” BMC Medical Genetics 20, no. 1: 39. 10.1186/s12881-019-0764-4. 30866842 PMC6417232

[jdn70042-bib-0146] Walters, H. , and T. A. Kosten . 2019. “Early Life Stress and the Propensity to Develop Addictive Behaviors.” International Journal of Environmental Research and Public Health 78: 156–169. 10.1016/j.ijdevneu.2019.06.004. 31255718

[jdn70042-bib-0147] Wang, J. , G. E. Hodes , H. Zhang , S. Zhang , W. Zhao , and S. A. Golden . 2018. “Epigenetic Modulation of Inflammation and Synaptic Plasticity Promotes Resilience Against Stress in Mice.” Nature Communications 9, no. 1: 1–14. 10.1038/s41467-017-02794-5. PMC5797143 29396460

[jdn70042-bib-0148] Wheeler, R. V. 2019. “The Importance of the Epigenome for Social‐Related Neural Circuits.” Frontiers in Neuroscience 7: 144. 10.2217/epi-2019-0255. 31701758

[jdn70042-bib-0149] Yehuda, R. , N. P. Daskalakis , F. Desarnaud , et al. 2013. “Epigenetic Biomarkers as Predictors and Correlates of Symptom Improvement Following Psychotherapy in Combat Veterans With PTSD.” Frontiers in Psychiatry 4: 118. 10.3389/fpsyt.2013.00118. 24098286 PMC3784793

[jdn70042-bib-0150] Zannas, A. S. , J. Arloth , T. Carrillo‐Roa , et al. 2015. “Lifetime Stress Accelerates Epigenetic Aging in an Urban, African American Cohort: Relevance of Glucocorticoid Signaling.” Genome Biology 16: 266. 10.1186/s13059-015-0828-5. 26673150 PMC4699359

[jdn70042-bib-0151] Zannas, A. S. , N. Provençal , and E. B. Binder . 2015. “Epigenetics of Posttraumatic Stress Disorder: Current Evidence, Challenges, and Future Directions.” Biological Psychiatry 78, no. 5: 327–335. PMID: 25890409. 10.1016/j.biopsych.2015.04.003. 25979620

[jdn70042-bib-0152] Zannas, A. S. , and A. E. West . 2013. “Epigenetics and the Regulation of Stress Vulnerability and Resilience.” Neuroscience 264: 157–170. 10.1016/j.neuroscience.2013.12.003. 24333971 PMC3959582

[jdn70042-bib-0153] Zovkik, I. B. , J. P. Meadows , G. A. Kaas , and J. D. Sweatt . 2013. “Interindividual Variability in Stress Susceptibility: A Role for Epigenetic Mechanisms in PTSD.” Frontiers in Psychiatry 4: 60. 23805109 10.3389/fpsyt.2013.00060 PMC3693073

